# Supplementary figures and images for: Small facial image dataset augmentation using conditional GANs based on incomplete edge feature input (part 1 of 6)
Source: PeerJ Comput Sci. 2021 Nov 17;7:e760. doi: 10.7717/peerj-cs.760 (PMC8627232; doi:10.7717/peerj-cs.760)

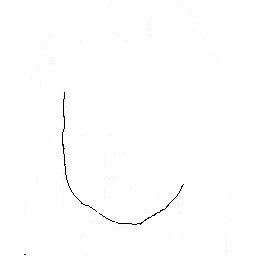

Supplement: Supplemental Information 2 [file peerj-cs-07-760-s002.zip › augmented facial images with hand drawing lines/inputs/13 - Copy (2)-inputs.png]

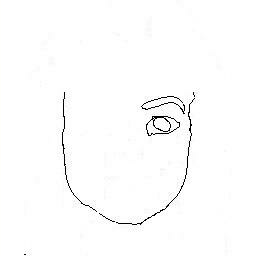

Supplement: Supplemental Information 2 [file peerj-cs-07-760-s002.zip › augmented facial images with hand drawing lines/inputs/13 - Copy (3)-inputs.png]

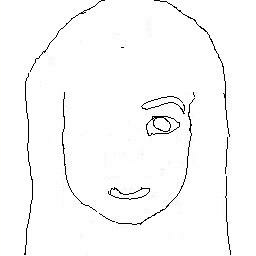

Supplement: Supplemental Information 2 [file peerj-cs-07-760-s002.zip › augmented facial images with hand drawing lines/inputs/13 - Copy (4)-inputs.png]

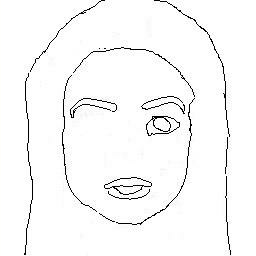

Supplement: Supplemental Information 2 [file peerj-cs-07-760-s002.zip › augmented facial images with hand drawing lines/inputs/13 - Copy (5)-inputs.png]

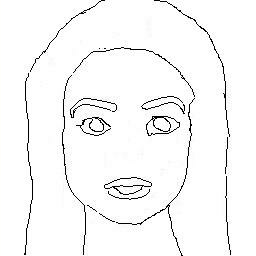

Supplement: Supplemental Information 2 [file peerj-cs-07-760-s002.zip › augmented facial images with hand drawing lines/inputs/13 - Copy-inputs.png]

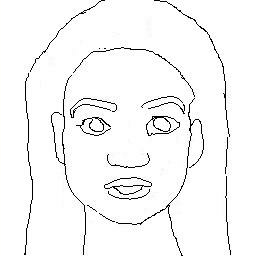

Supplement: Supplemental Information 2 [file peerj-cs-07-760-s002.zip › augmented facial images with hand drawing lines/inputs/13-inputs.png]

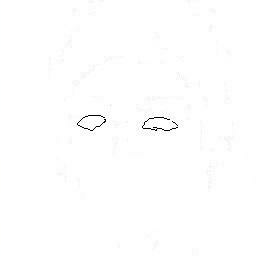

Supplement: Supplemental Information 2 [file peerj-cs-07-760-s002.zip › augmented facial images with hand drawing lines/inputs/18 - Copy (2)-inputs.png]

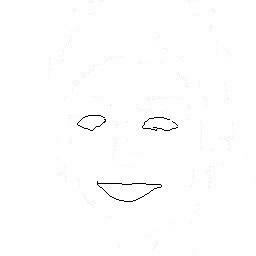

Supplement: Supplemental Information 2 [file peerj-cs-07-760-s002.zip › augmented facial images with hand drawing lines/inputs/18 - Copy (3)-inputs.png]

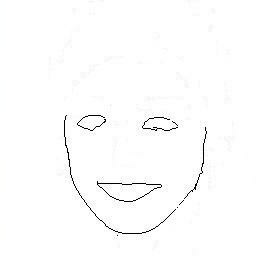

Supplement: Supplemental Information 2 [file peerj-cs-07-760-s002.zip › augmented facial images with hand drawing lines/inputs/18 - Copy (4)-inputs.png]

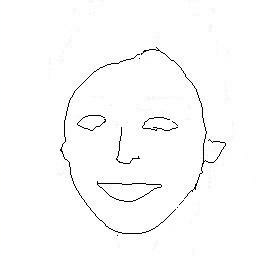

Supplement: Supplemental Information 2 [file peerj-cs-07-760-s002.zip › augmented facial images with hand drawing lines/inputs/18 - Copy (5)-inputs.png]

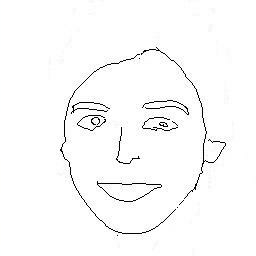

Supplement: Supplemental Information 2 [file peerj-cs-07-760-s002.zip › augmented facial images with hand drawing lines/inputs/18 - Copy-inputs.png]

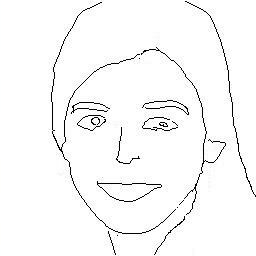

Supplement: Supplemental Information 2 [file peerj-cs-07-760-s002.zip › augmented facial images with hand drawing lines/inputs/18-inputs.png]

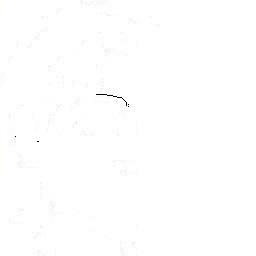

Supplement: Supplemental Information 2 [file peerj-cs-07-760-s002.zip › augmented facial images with hand drawing lines/inputs/19 - Copy (2)-inputs.png]

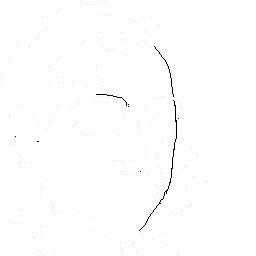

Supplement: Supplemental Information 2 [file peerj-cs-07-760-s002.zip › augmented facial images with hand drawing lines/inputs/19 - Copy (3)-inputs.png]

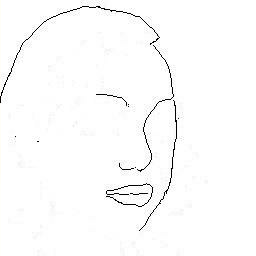

Supplement: Supplemental Information 2 [file peerj-cs-07-760-s002.zip › augmented facial images with hand drawing lines/inputs/19 - Copy (4)-inputs.png]

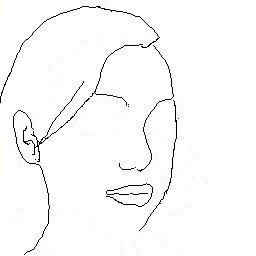

Supplement: Supplemental Information 2 [file peerj-cs-07-760-s002.zip › augmented facial images with hand drawing lines/inputs/19 - Copy (5)-inputs.png]

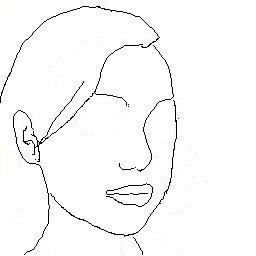

Supplement: Supplemental Information 2 [file peerj-cs-07-760-s002.zip › augmented facial images with hand drawing lines/inputs/19 - Copy-inputs.png]

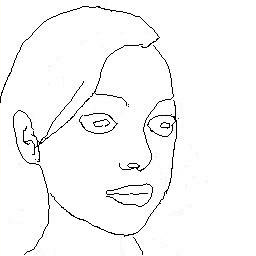

Supplement: Supplemental Information 2 [file peerj-cs-07-760-s002.zip › augmented facial images with hand drawing lines/inputs/19-inputs.png]

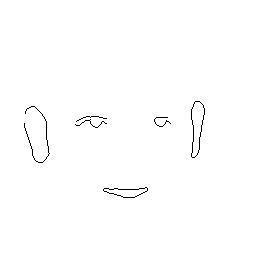

Supplement: Supplemental Information 2 [file peerj-cs-07-760-s002.zip › augmented facial images with hand drawing lines/inputs/28837 - Copy (2)-inputs.png]

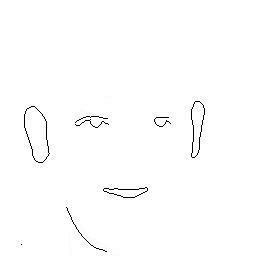

Supplement: Supplemental Information 2 [file peerj-cs-07-760-s002.zip › augmented facial images with hand drawing lines/inputs/28837 - Copy (3)-inputs.png]

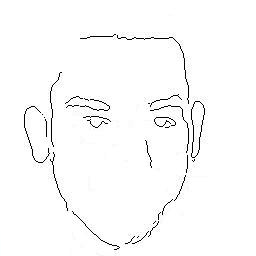

Supplement: Supplemental Information 2 [file peerj-cs-07-760-s002.zip › augmented facial images with hand drawing lines/inputs/28837 - Copy (4)-inputs.png]

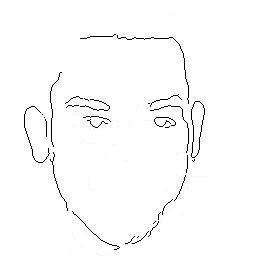

Supplement: Supplemental Information 2 [file peerj-cs-07-760-s002.zip › augmented facial images with hand drawing lines/inputs/28837 - Copy (5)-inputs.png]

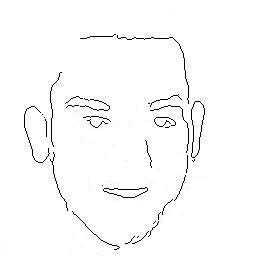

Supplement: Supplemental Information 2 [file peerj-cs-07-760-s002.zip › augmented facial images with hand drawing lines/inputs/28837 - Copy-inputs.png]

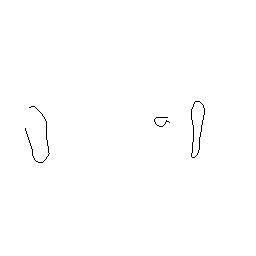

Supplement: Supplemental Information 2 [file peerj-cs-07-760-s002.zip › augmented facial images with hand drawing lines/inputs/28837-inputs.png]

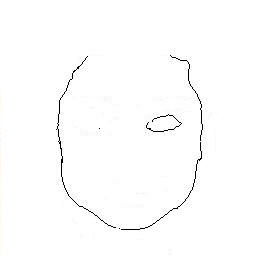

Supplement: Supplemental Information 2 [file peerj-cs-07-760-s002.zip › augmented facial images with hand drawing lines/inputs/29105 - Copy (2)-inputs.png]

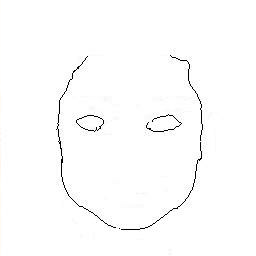

Supplement: Supplemental Information 2 [file peerj-cs-07-760-s002.zip › augmented facial images with hand drawing lines/inputs/29105 - Copy (3)-inputs.png]

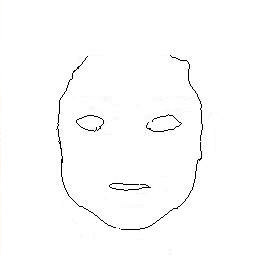

Supplement: Supplemental Information 2 [file peerj-cs-07-760-s002.zip › augmented facial images with hand drawing lines/inputs/29105 - Copy (4)-inputs.png]

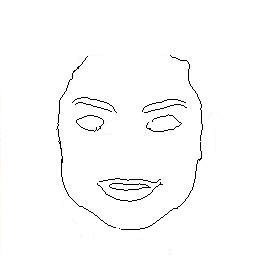

Supplement: Supplemental Information 2 [file peerj-cs-07-760-s002.zip › augmented facial images with hand drawing lines/inputs/29105 - Copy (5)-inputs.png]

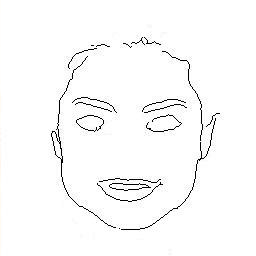

Supplement: Supplemental Information 2 [file peerj-cs-07-760-s002.zip › augmented facial images with hand drawing lines/inputs/29105 - Copy-inputs.png]

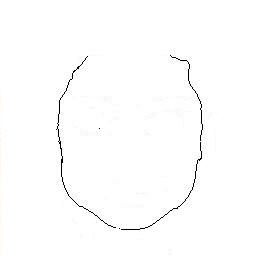

Supplement: Supplemental Information 2 [file peerj-cs-07-760-s002.zip › augmented facial images with hand drawing lines/inputs/29105-inputs.png]

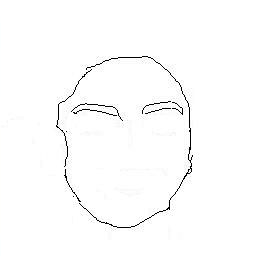

Supplement: Supplemental Information 2 [file peerj-cs-07-760-s002.zip › augmented facial images with hand drawing lines/inputs/29174 - Copy (2)-inputs.png]

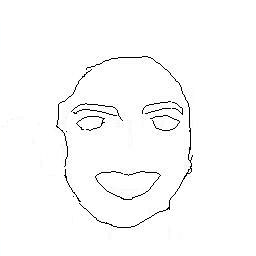

Supplement: Supplemental Information 2 [file peerj-cs-07-760-s002.zip › augmented facial images with hand drawing lines/inputs/29174 - Copy (3)-inputs.png]

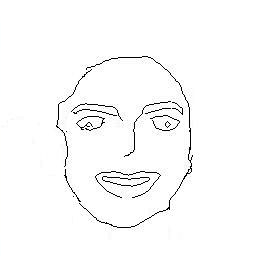

Supplement: Supplemental Information 2 [file peerj-cs-07-760-s002.zip › augmented facial images with hand drawing lines/inputs/29174 - Copy (4)-inputs.png]

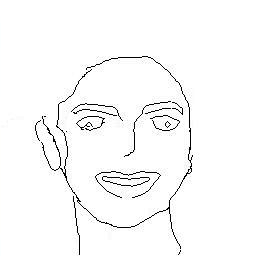

Supplement: Supplemental Information 2 [file peerj-cs-07-760-s002.zip › augmented facial images with hand drawing lines/inputs/29174 - Copy (5)-inputs.png]

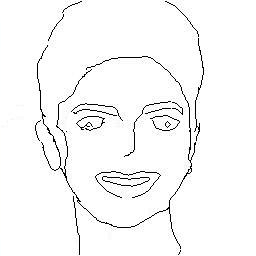

Supplement: Supplemental Information 2 [file peerj-cs-07-760-s002.zip › augmented facial images with hand drawing lines/inputs/29174 - Copy-inputs.png]

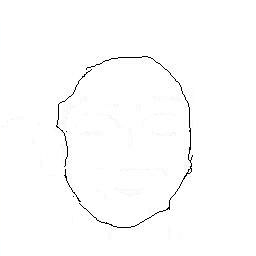

Supplement: Supplemental Information 2 [file peerj-cs-07-760-s002.zip › augmented facial images with hand drawing lines/inputs/29174-inputs.png]

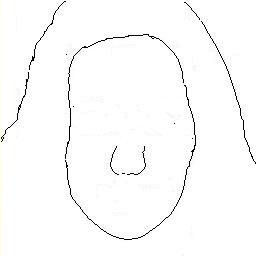

Supplement: Supplemental Information 2 [file peerj-cs-07-760-s002.zip › augmented facial images with hand drawing lines/inputs/29185 - Copy (2)-inputs.png]

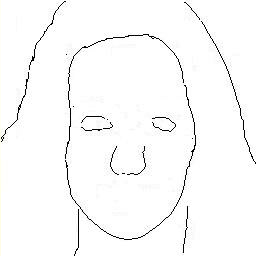

Supplement: Supplemental Information 2 [file peerj-cs-07-760-s002.zip › augmented facial images with hand drawing lines/inputs/29185 - Copy (3)-inputs.png]

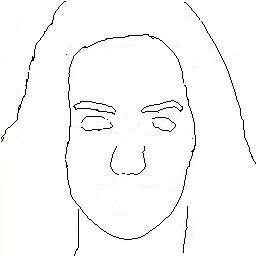

Supplement: Supplemental Information 2 [file peerj-cs-07-760-s002.zip › augmented facial images with hand drawing lines/inputs/29185 - Copy (4)-inputs.png]

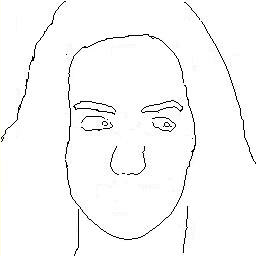

Supplement: Supplemental Information 2 [file peerj-cs-07-760-s002.zip › augmented facial images with hand drawing lines/inputs/29185 - Copy (5)-inputs.png]

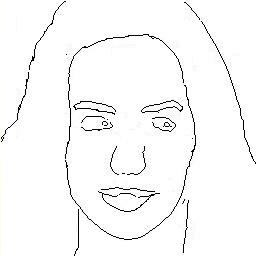

Supplement: Supplemental Information 2 [file peerj-cs-07-760-s002.zip › augmented facial images with hand drawing lines/inputs/29185 - Copy-inputs.png]

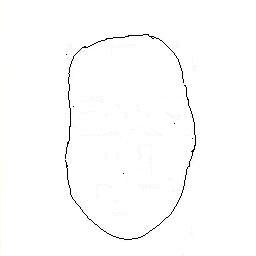

Supplement: Supplemental Information 2 [file peerj-cs-07-760-s002.zip › augmented facial images with hand drawing lines/inputs/29185-inputs.png]

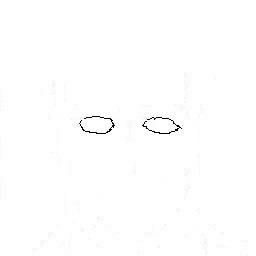

Supplement: Supplemental Information 2 [file peerj-cs-07-760-s002.zip › augmented facial images with hand drawing lines/inputs/39 - Copy (2)-inputs.png]

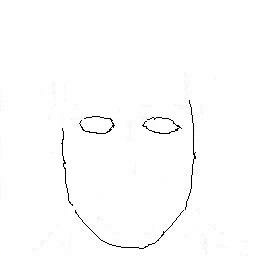

Supplement: Supplemental Information 2 [file peerj-cs-07-760-s002.zip › augmented facial images with hand drawing lines/inputs/39 - Copy (3)-inputs.png]

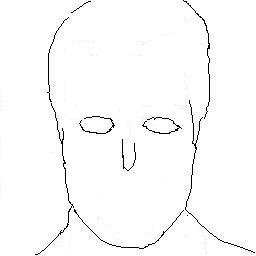

Supplement: Supplemental Information 2 [file peerj-cs-07-760-s002.zip › augmented facial images with hand drawing lines/inputs/39 - Copy (4)-inputs.png]

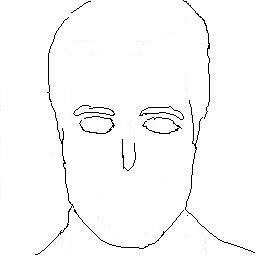

Supplement: Supplemental Information 2 [file peerj-cs-07-760-s002.zip › augmented facial images with hand drawing lines/inputs/39 - Copy (5)-inputs.png]

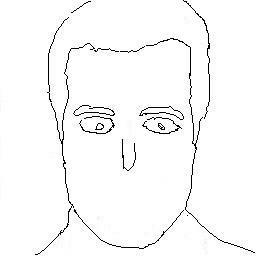

Supplement: Supplemental Information 2 [file peerj-cs-07-760-s002.zip › augmented facial images with hand drawing lines/inputs/39 - Copy-inputs.png]

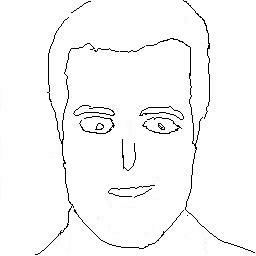

Supplement: Supplemental Information 2 [file peerj-cs-07-760-s002.zip › augmented facial images with hand drawing lines/inputs/39-inputs.png]

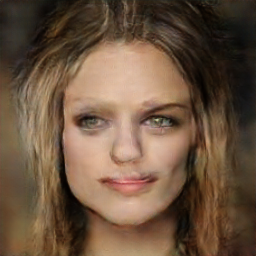

Supplement: Supplemental Information 2 [file peerj-cs-07-760-s002.zip › augmented facial images with hand drawing lines/outputs/13 - Copy (2)-targets-outputs.png]

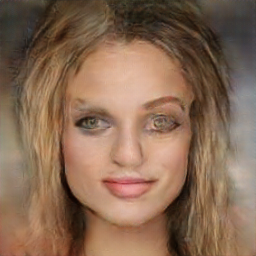

Supplement: Supplemental Information 2 [file peerj-cs-07-760-s002.zip › augmented facial images with hand drawing lines/outputs/13 - Copy (3)-targets-outputs.png]

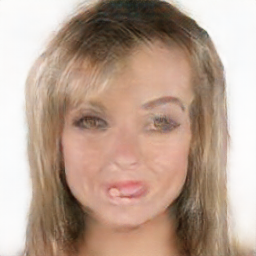

Supplement: Supplemental Information 2 [file peerj-cs-07-760-s002.zip › augmented facial images with hand drawing lines/outputs/13 - Copy (4)-targets-outputs.png]

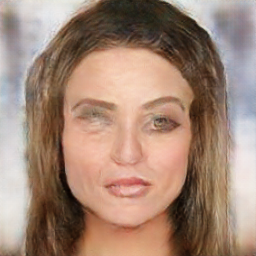

Supplement: Supplemental Information 2 [file peerj-cs-07-760-s002.zip › augmented facial images with hand drawing lines/outputs/13 - Copy (5)-targets-outputs.png]

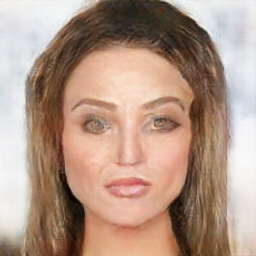

Supplement: Supplemental Information 2 [file peerj-cs-07-760-s002.zip › augmented facial images with hand drawing lines/outputs/13 - Copy-targets-outputs.png]

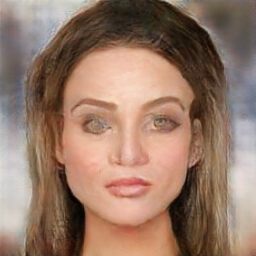

Supplement: Supplemental Information 2 [file peerj-cs-07-760-s002.zip › augmented facial images with hand drawing lines/outputs/13-targets-outputs.png]

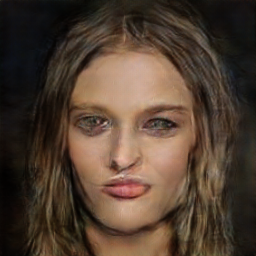

Supplement: Supplemental Information 2 [file peerj-cs-07-760-s002.zip › augmented facial images with hand drawing lines/outputs/18 - Copy (2)-targets-outputs.png]

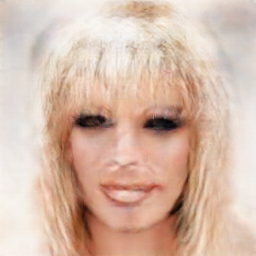

Supplement: Supplemental Information 2 [file peerj-cs-07-760-s002.zip › augmented facial images with hand drawing lines/outputs/18 - Copy (3)-targets-outputs.png]

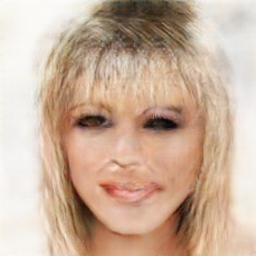

Supplement: Supplemental Information 2 [file peerj-cs-07-760-s002.zip › augmented facial images with hand drawing lines/outputs/18 - Copy (4)-targets-outputs.png]

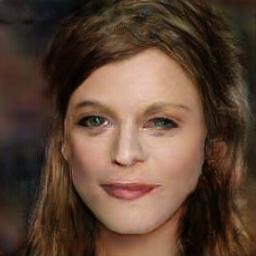

Supplement: Supplemental Information 2 [file peerj-cs-07-760-s002.zip › augmented facial images with hand drawing lines/outputs/18 - Copy (5)-targets-outputs.png]

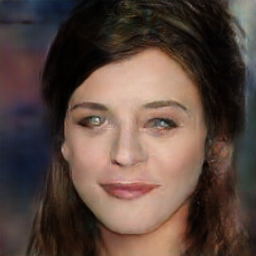

Supplement: Supplemental Information 2 [file peerj-cs-07-760-s002.zip › augmented facial images with hand drawing lines/outputs/18 - Copy-targets-outputs.png]

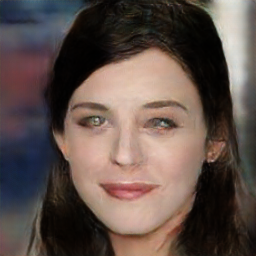

Supplement: Supplemental Information 2 [file peerj-cs-07-760-s002.zip › augmented facial images with hand drawing lines/outputs/18-targets-outputs.png]

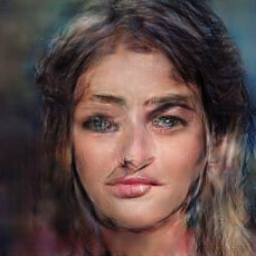

Supplement: Supplemental Information 2 [file peerj-cs-07-760-s002.zip › augmented facial images with hand drawing lines/outputs/19 - Copy (2)-targets-outputs.png]

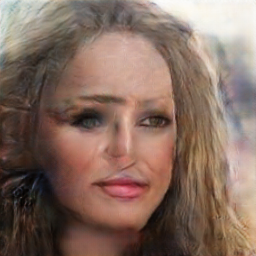

Supplement: Supplemental Information 2 [file peerj-cs-07-760-s002.zip › augmented facial images with hand drawing lines/outputs/19 - Copy (3)-targets-outputs.png]

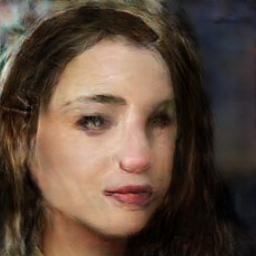

Supplement: Supplemental Information 2 [file peerj-cs-07-760-s002.zip › augmented facial images with hand drawing lines/outputs/19 - Copy (4)-targets-outputs.png]

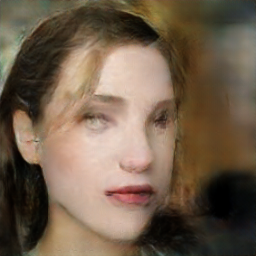

Supplement: Supplemental Information 2 [file peerj-cs-07-760-s002.zip › augmented facial images with hand drawing lines/outputs/19 - Copy (5)-targets-outputs.png]

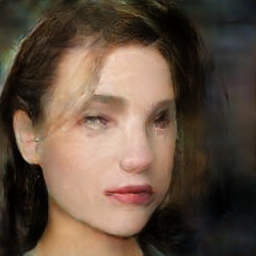

Supplement: Supplemental Information 2 [file peerj-cs-07-760-s002.zip › augmented facial images with hand drawing lines/outputs/19 - Copy-targets-outputs.png]

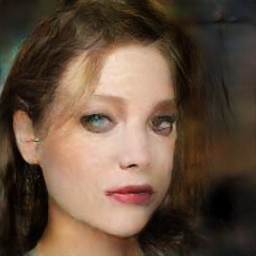

Supplement: Supplemental Information 2 [file peerj-cs-07-760-s002.zip › augmented facial images with hand drawing lines/outputs/19-targets-outputs.png]

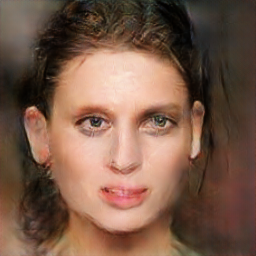

Supplement: Supplemental Information 2 [file peerj-cs-07-760-s002.zip › augmented facial images with hand drawing lines/outputs/28837 - Copy (2)-targets-outputs.png]

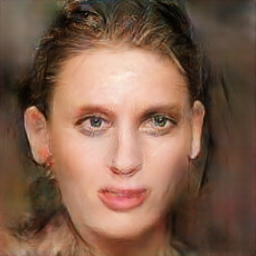

Supplement: Supplemental Information 2 [file peerj-cs-07-760-s002.zip › augmented facial images with hand drawing lines/outputs/28837 - Copy (3)-targets-outputs.png]

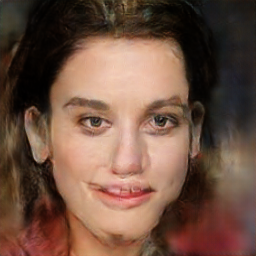

Supplement: Supplemental Information 2 [file peerj-cs-07-760-s002.zip › augmented facial images with hand drawing lines/outputs/28837 - Copy (4)-targets-outputs.png]

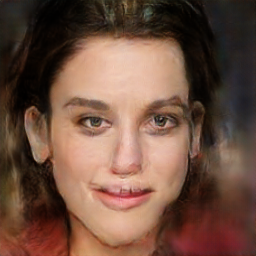

Supplement: Supplemental Information 2 [file peerj-cs-07-760-s002.zip › augmented facial images with hand drawing lines/outputs/28837 - Copy (5)-targets-outputs.png]

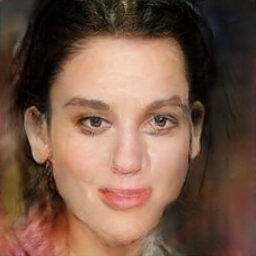

Supplement: Supplemental Information 2 [file peerj-cs-07-760-s002.zip › augmented facial images with hand drawing lines/outputs/28837 - Copy-targets-outputs.png]

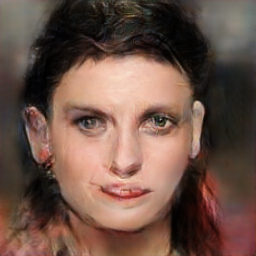

Supplement: Supplemental Information 2 [file peerj-cs-07-760-s002.zip › augmented facial images with hand drawing lines/outputs/28837-targets-outputs.png]

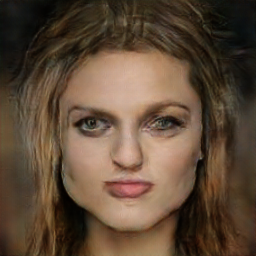

Supplement: Supplemental Information 2 [file peerj-cs-07-760-s002.zip › augmented facial images with hand drawing lines/outputs/29105 - Copy (2)-targets-outputs.png]

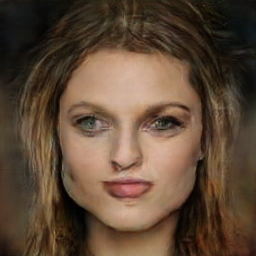

Supplement: Supplemental Information 2 [file peerj-cs-07-760-s002.zip › augmented facial images with hand drawing lines/outputs/29105 - Copy (3)-targets-outputs.png]

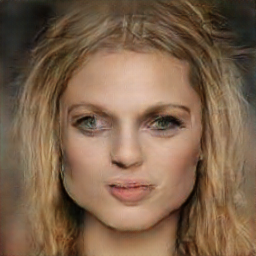

Supplement: Supplemental Information 2 [file peerj-cs-07-760-s002.zip › augmented facial images with hand drawing lines/outputs/29105 - Copy (4)-targets-outputs.png]

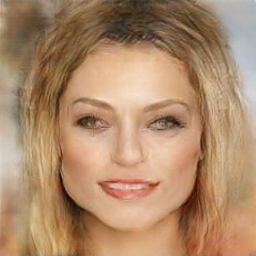

Supplement: Supplemental Information 2 [file peerj-cs-07-760-s002.zip › augmented facial images with hand drawing lines/outputs/29105 - Copy (5)-targets-outputs.png]

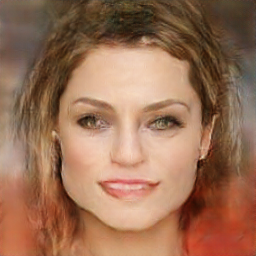

Supplement: Supplemental Information 2 [file peerj-cs-07-760-s002.zip › augmented facial images with hand drawing lines/outputs/29105 - Copy-targets-outputs.png]

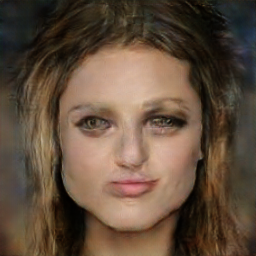

Supplement: Supplemental Information 2 [file peerj-cs-07-760-s002.zip › augmented facial images with hand drawing lines/outputs/29105-targets-outputs.png]

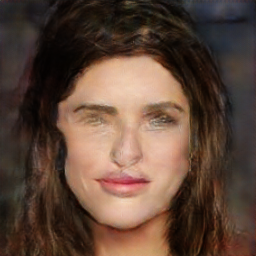

Supplement: Supplemental Information 2 [file peerj-cs-07-760-s002.zip › augmented facial images with hand drawing lines/outputs/29174 - Copy (2)-targets-outputs.png]

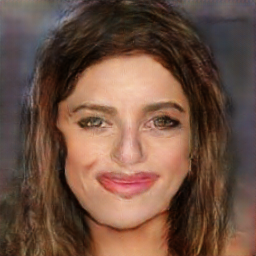

Supplement: Supplemental Information 2 [file peerj-cs-07-760-s002.zip › augmented facial images with hand drawing lines/outputs/29174 - Copy (3)-targets-outputs.png]

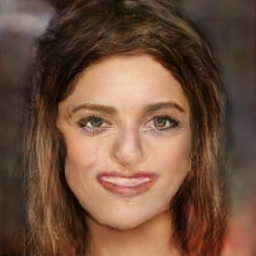

Supplement: Supplemental Information 2 [file peerj-cs-07-760-s002.zip › augmented facial images with hand drawing lines/outputs/29174 - Copy (4)-targets-outputs.png]

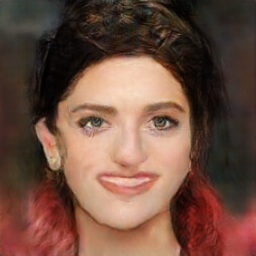

Supplement: Supplemental Information 2 [file peerj-cs-07-760-s002.zip › augmented facial images with hand drawing lines/outputs/29174 - Copy (5)-targets-outputs.png]

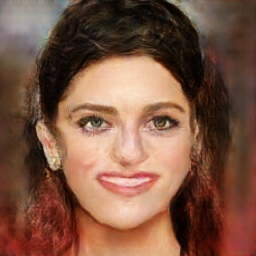

Supplement: Supplemental Information 2 [file peerj-cs-07-760-s002.zip › augmented facial images with hand drawing lines/outputs/29174 - Copy-targets-outputs.png]

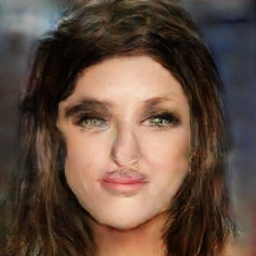

Supplement: Supplemental Information 2 [file peerj-cs-07-760-s002.zip › augmented facial images with hand drawing lines/outputs/29174-targets-outputs.png]

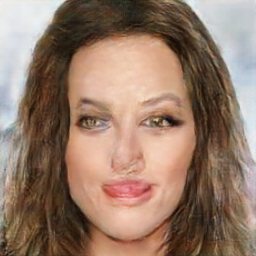

Supplement: Supplemental Information 2 [file peerj-cs-07-760-s002.zip › augmented facial images with hand drawing lines/outputs/29185 - Copy (2)-targets-outputs.png]

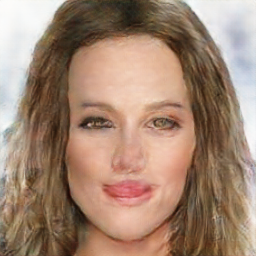

Supplement: Supplemental Information 2 [file peerj-cs-07-760-s002.zip › augmented facial images with hand drawing lines/outputs/29185 - Copy (3)-targets-outputs.png]

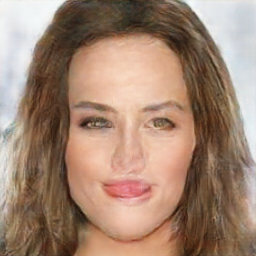

Supplement: Supplemental Information 2 [file peerj-cs-07-760-s002.zip › augmented facial images with hand drawing lines/outputs/29185 - Copy (4)-targets-outputs.png]

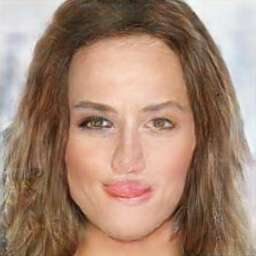

Supplement: Supplemental Information 2 [file peerj-cs-07-760-s002.zip › augmented facial images with hand drawing lines/outputs/29185 - Copy (5)-targets-outputs.png]

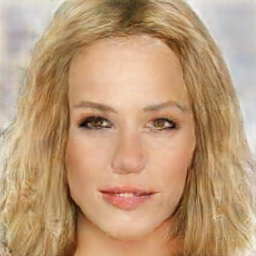

Supplement: Supplemental Information 2 [file peerj-cs-07-760-s002.zip › augmented facial images with hand drawing lines/outputs/29185 - Copy-targets-outputs.png]

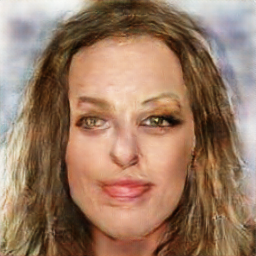

Supplement: Supplemental Information 2 [file peerj-cs-07-760-s002.zip › augmented facial images with hand drawing lines/outputs/29185-targets-outputs.png]

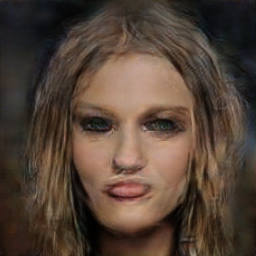

Supplement: Supplemental Information 2 [file peerj-cs-07-760-s002.zip › augmented facial images with hand drawing lines/outputs/39 - Copy (2)-targets-outputs.png]

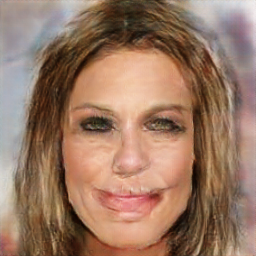

Supplement: Supplemental Information 2 [file peerj-cs-07-760-s002.zip › augmented facial images with hand drawing lines/outputs/39 - Copy (3)-targets-outputs.png]

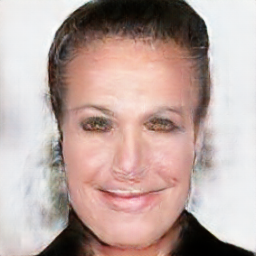

Supplement: Supplemental Information 2 [file peerj-cs-07-760-s002.zip › augmented facial images with hand drawing lines/outputs/39 - Copy (4)-targets-outputs.png]

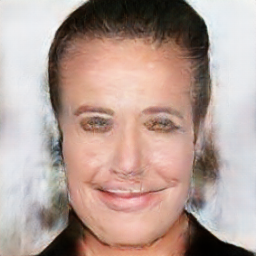

Supplement: Supplemental Information 2 [file peerj-cs-07-760-s002.zip › augmented facial images with hand drawing lines/outputs/39 - Copy (5)-targets-outputs.png]

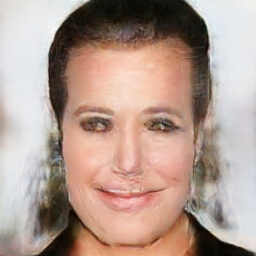

Supplement: Supplemental Information 2 [file peerj-cs-07-760-s002.zip › augmented facial images with hand drawing lines/outputs/39 - Copy-targets-outputs.png]

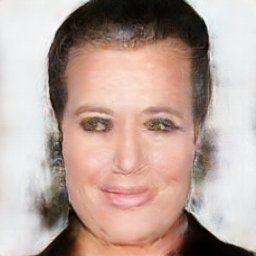

Supplement: Supplemental Information 2 [file peerj-cs-07-760-s002.zip › augmented facial images with hand drawing lines/outputs/39-targets-outputs.png]

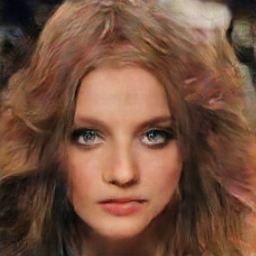

Supplement: Supplemental Information 3 [file peerj-cs-07-760-s003.zip › augmented facial images with sparse lines/single image blending/12-10-targets-outputs.png]

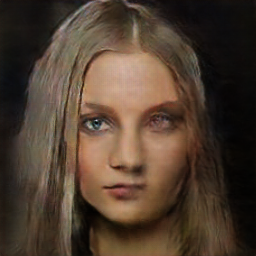

Supplement: Supplemental Information 3 [file peerj-cs-07-760-s003.zip › augmented facial images with sparse lines/single image blending/12-11-targets-outputs.png]

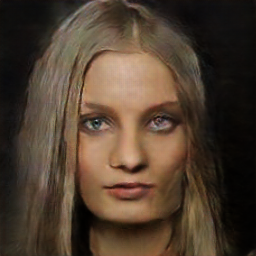

Supplement: Supplemental Information 3 [file peerj-cs-07-760-s003.zip › augmented facial images with sparse lines/single image blending/12-1-targets-outputs.png]

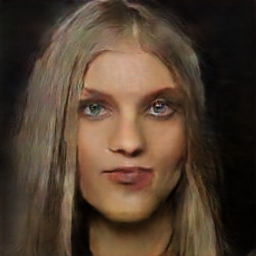

Supplement: Supplemental Information 3 [file peerj-cs-07-760-s003.zip › augmented facial images with sparse lines/single image blending/12-2-targets-outputs.png]
