# Supplementary figures and images for: Small facial image dataset augmentation using conditional GANs based on incomplete edge feature input (part 3 of 6)
Source: PeerJ Comput Sci. 2021 Nov 17;7:e760. doi: 10.7717/peerj-cs.760 (PMC8627232; doi:10.7717/peerj-cs.760)

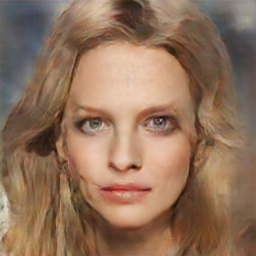

Supplement: Supplemental Information 3 [file peerj-cs-07-760-s003.zip › augmented facial images with sparse lines/two image swap/48-20-targets-outputs.png]

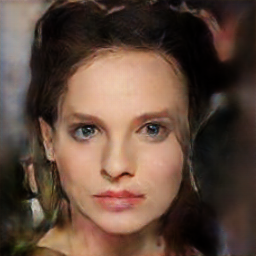

Supplement: Supplemental Information 3 [file peerj-cs-07-760-s003.zip › augmented facial images with sparse lines/two image swap/48-27-targets-outputs.png]

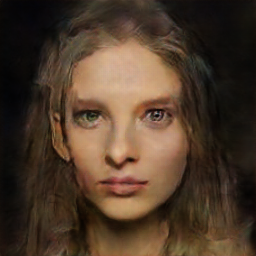

Supplement: Supplemental Information 3 [file peerj-cs-07-760-s003.zip › augmented facial images with sparse lines/two image swap/48-42-targets-outputs.png]

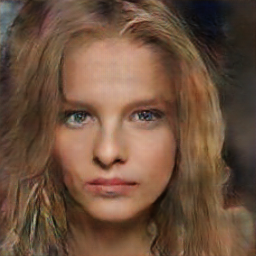

Supplement: Supplemental Information 3 [file peerj-cs-07-760-s003.zip › augmented facial images with sparse lines/two image swap/48-46-targets-outputs.png]

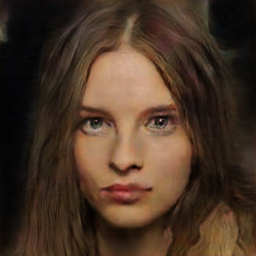

Supplement: Supplemental Information 3 [file peerj-cs-07-760-s003.zip › augmented facial images with sparse lines/two image swap/48-targets-outputs.png]

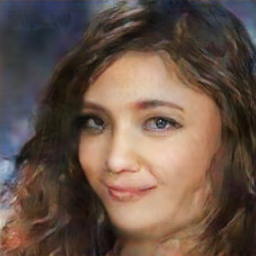

Supplement: Supplemental Information 4 [file peerj-cs-07-760-s004.zip › 01/201-targets-outputs.png]

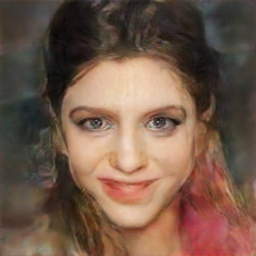

Supplement: Supplemental Information 4 [file peerj-cs-07-760-s004.zip › 01/202-targets-outputs.png]

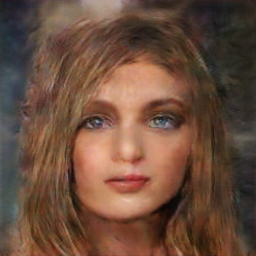

Supplement: Supplemental Information 4 [file peerj-cs-07-760-s004.zip › 01/203-targets-outputs.png]

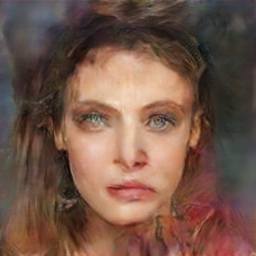

Supplement: Supplemental Information 4 [file peerj-cs-07-760-s004.zip › 01/204-targets-outputs.png]

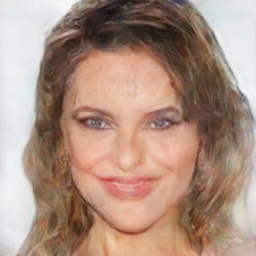

Supplement: Supplemental Information 4 [file peerj-cs-07-760-s004.zip › 01/205-targets-outputs.png]

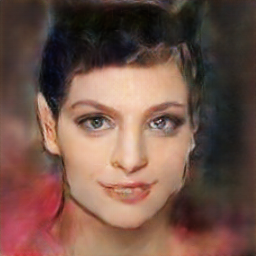

Supplement: Supplemental Information 4 [file peerj-cs-07-760-s004.zip › 01/206-targets-outputs.png]

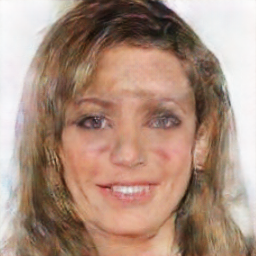

Supplement: Supplemental Information 4 [file peerj-cs-07-760-s004.zip › 01/207-targets-outputs.png]

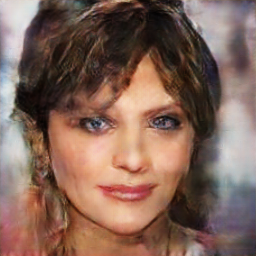

Supplement: Supplemental Information 4 [file peerj-cs-07-760-s004.zip › 01/208-targets-outputs.png]

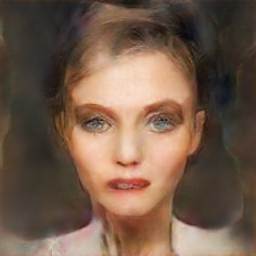

Supplement: Supplemental Information 4 [file peerj-cs-07-760-s004.zip › 01/209-targets-outputs.png]

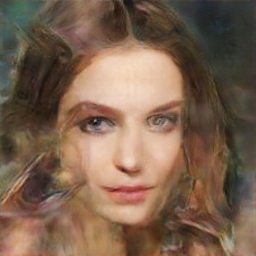

Supplement: Supplemental Information 4 [file peerj-cs-07-760-s004.zip › 01/210-targets-outputs.png]

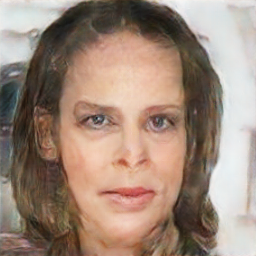

Supplement: Supplemental Information 4 [file peerj-cs-07-760-s004.zip › 01/211-targets-outputs.png]

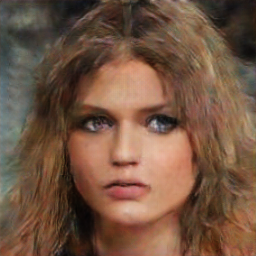

Supplement: Supplemental Information 4 [file peerj-cs-07-760-s004.zip › 01/212-targets-outputs.png]

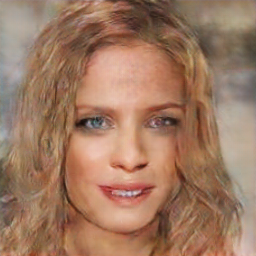

Supplement: Supplemental Information 4 [file peerj-cs-07-760-s004.zip › 01/213-targets-outputs.png]

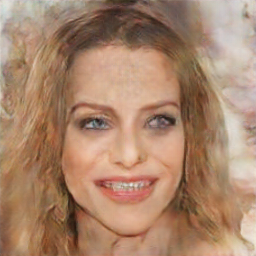

Supplement: Supplemental Information 4 [file peerj-cs-07-760-s004.zip › 01/214-targets-outputs.png]

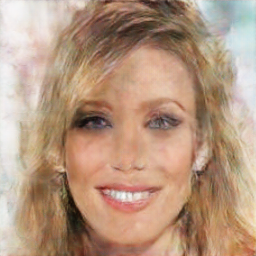

Supplement: Supplemental Information 4 [file peerj-cs-07-760-s004.zip › 01/215-targets-outputs.png]

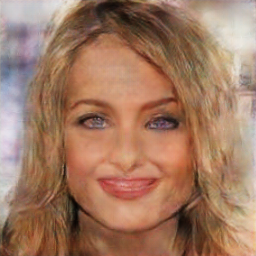

Supplement: Supplemental Information 4 [file peerj-cs-07-760-s004.zip › 01/216-targets-outputs.png]

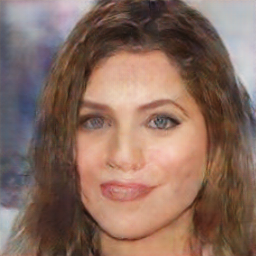

Supplement: Supplemental Information 4 [file peerj-cs-07-760-s004.zip › 01/217-targets-outputs.png]

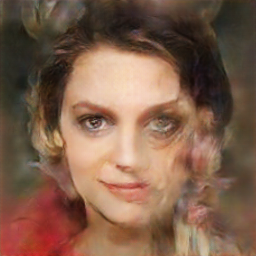

Supplement: Supplemental Information 4 [file peerj-cs-07-760-s004.zip › 01/218-targets-outputs.png]

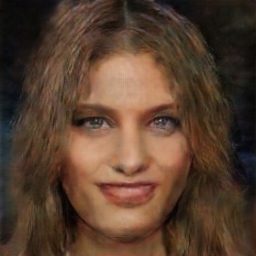

Supplement: Supplemental Information 4 [file peerj-cs-07-760-s004.zip › 01/219-targets-outputs.png]

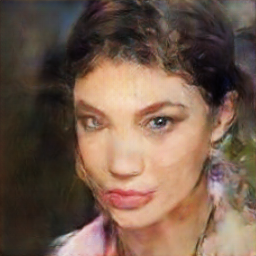

Supplement: Supplemental Information 4 [file peerj-cs-07-760-s004.zip › 01/220-targets-outputs.png]

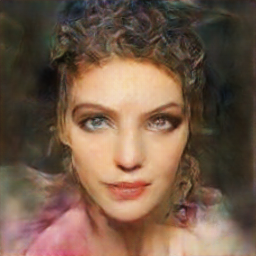

Supplement: Supplemental Information 4 [file peerj-cs-07-760-s004.zip › 01/221-targets-outputs.png]

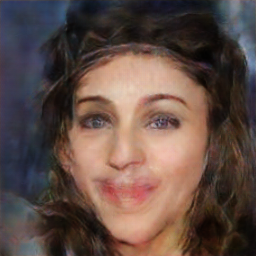

Supplement: Supplemental Information 4 [file peerj-cs-07-760-s004.zip › 01/222-targets-outputs.png]

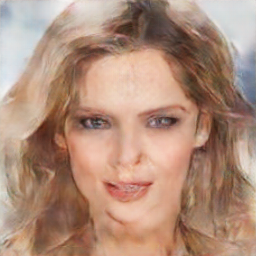

Supplement: Supplemental Information 4 [file peerj-cs-07-760-s004.zip › 01/223-targets-outputs.png]

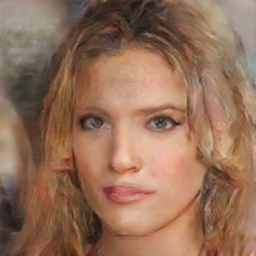

Supplement: Supplemental Information 4 [file peerj-cs-07-760-s004.zip › 01/224-targets-outputs.png]

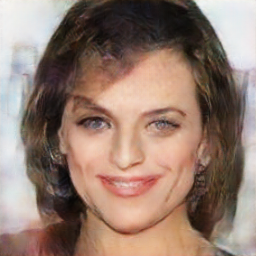

Supplement: Supplemental Information 4 [file peerj-cs-07-760-s004.zip › 01/225-targets-outputs.png]

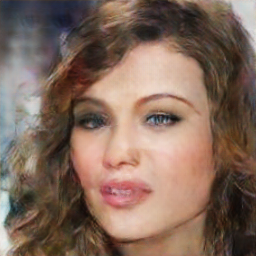

Supplement: Supplemental Information 4 [file peerj-cs-07-760-s004.zip › 01/226-targets-outputs.png]

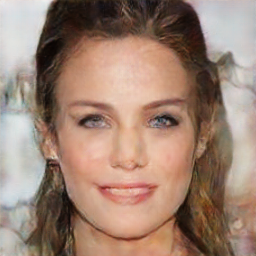

Supplement: Supplemental Information 4 [file peerj-cs-07-760-s004.zip › 01/227-targets-outputs.png]

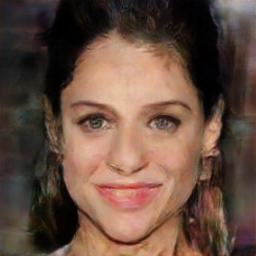

Supplement: Supplemental Information 4 [file peerj-cs-07-760-s004.zip › 01/228-targets-outputs.png]

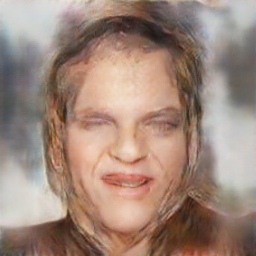

Supplement: Supplemental Information 4 [file peerj-cs-07-760-s004.zip › 01/229-targets-outputs.png]

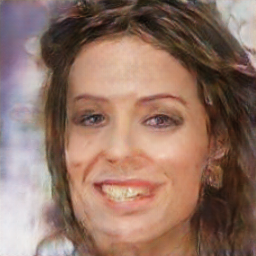

Supplement: Supplemental Information 4 [file peerj-cs-07-760-s004.zip › 01/230-targets-outputs.png]

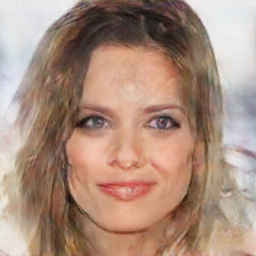

Supplement: Supplemental Information 4 [file peerj-cs-07-760-s004.zip › 01/231-targets-outputs.png]

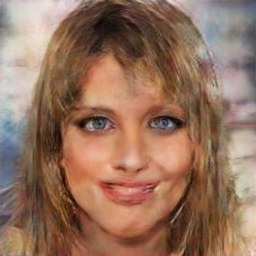

Supplement: Supplemental Information 4 [file peerj-cs-07-760-s004.zip › 01/232-targets-outputs.png]

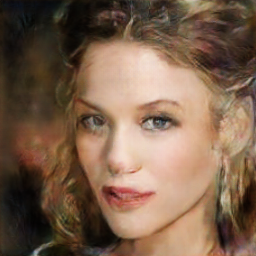

Supplement: Supplemental Information 4 [file peerj-cs-07-760-s004.zip › 01/233-targets-outputs.png]

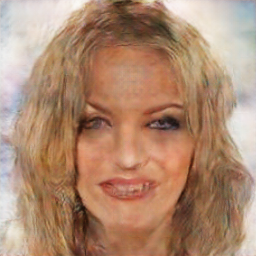

Supplement: Supplemental Information 4 [file peerj-cs-07-760-s004.zip › 01/234-targets-outputs.png]

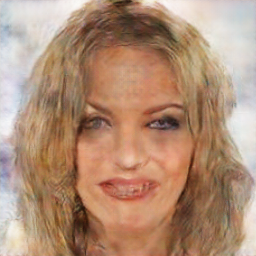

Supplement: Supplemental Information 4 [file peerj-cs-07-760-s004.zip › 01/235-targets-outputs.png]

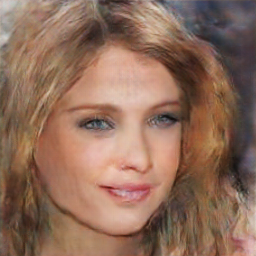

Supplement: Supplemental Information 4 [file peerj-cs-07-760-s004.zip › 01/236-targets-outputs.png]

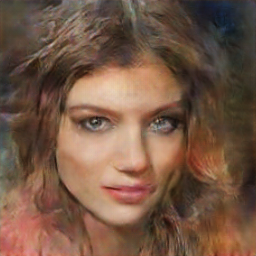

Supplement: Supplemental Information 4 [file peerj-cs-07-760-s004.zip › 01/237-targets-outputs.png]

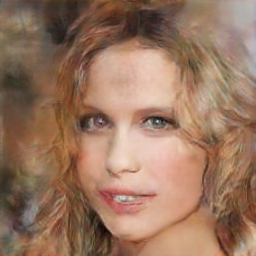

Supplement: Supplemental Information 4 [file peerj-cs-07-760-s004.zip › 01/238-targets-outputs.png]

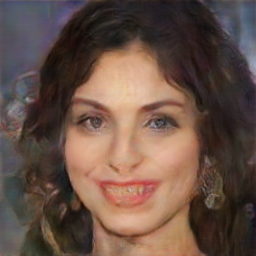

Supplement: Supplemental Information 4 [file peerj-cs-07-760-s004.zip › 01/239-targets-outputs.png]

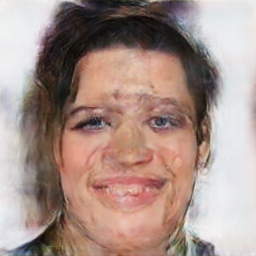

Supplement: Supplemental Information 4 [file peerj-cs-07-760-s004.zip › 01/240-targets-outputs.png]

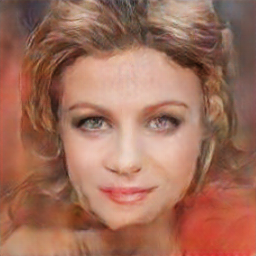

Supplement: Supplemental Information 4 [file peerj-cs-07-760-s004.zip › 01/241-targets-outputs.png]

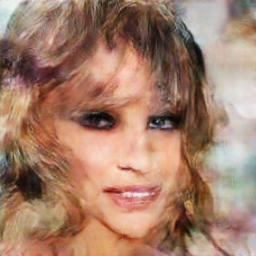

Supplement: Supplemental Information 4 [file peerj-cs-07-760-s004.zip › 01/242-targets-outputs.png]

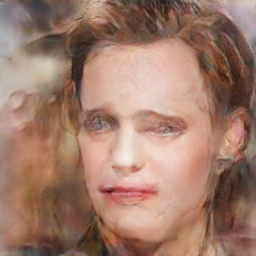

Supplement: Supplemental Information 4 [file peerj-cs-07-760-s004.zip › 01/243-targets-outputs.png]

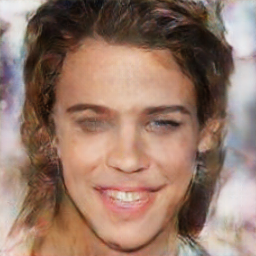

Supplement: Supplemental Information 4 [file peerj-cs-07-760-s004.zip › 01/244-targets-outputs.png]

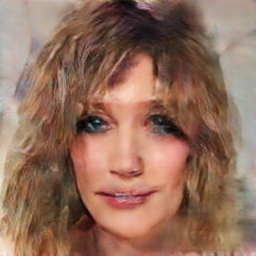

Supplement: Supplemental Information 4 [file peerj-cs-07-760-s004.zip › 01/245-targets-outputs.png]

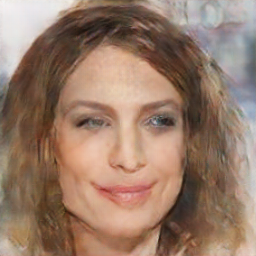

Supplement: Supplemental Information 4 [file peerj-cs-07-760-s004.zip › 01/246-targets-outputs.png]

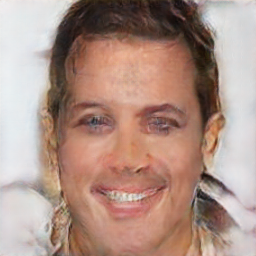

Supplement: Supplemental Information 4 [file peerj-cs-07-760-s004.zip › 01/247-targets-outputs.png]

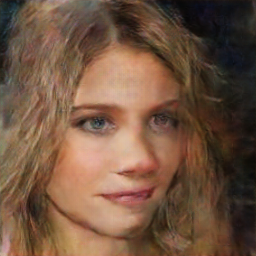

Supplement: Supplemental Information 4 [file peerj-cs-07-760-s004.zip › 01/248-targets-outputs.png]

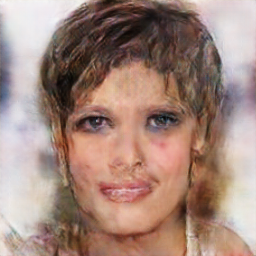

Supplement: Supplemental Information 4 [file peerj-cs-07-760-s004.zip › 01/249-targets-outputs.png]

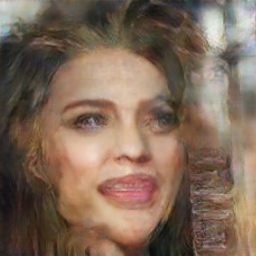

Supplement: Supplemental Information 4 [file peerj-cs-07-760-s004.zip › 01/250-targets-outputs.png]

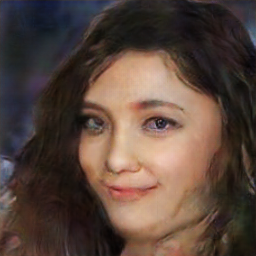

Supplement: Supplemental Information 4 [file peerj-cs-07-760-s004.zip › 02/201-targets-outputs.png]

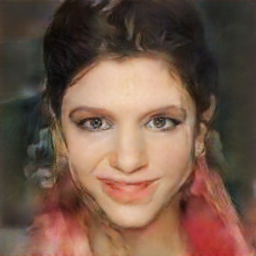

Supplement: Supplemental Information 4 [file peerj-cs-07-760-s004.zip › 02/202-targets-outputs.png]

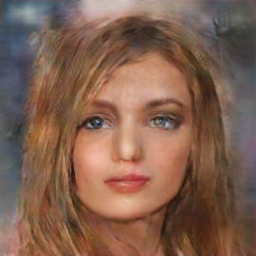

Supplement: Supplemental Information 4 [file peerj-cs-07-760-s004.zip › 02/203-targets-outputs.png]

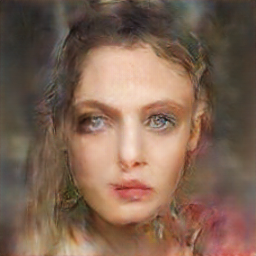

Supplement: Supplemental Information 4 [file peerj-cs-07-760-s004.zip › 02/204-targets-outputs.png]

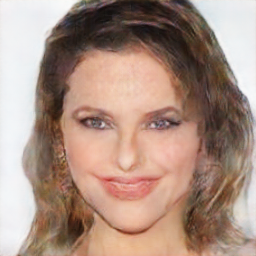

Supplement: Supplemental Information 4 [file peerj-cs-07-760-s004.zip › 02/205-targets-outputs.png]

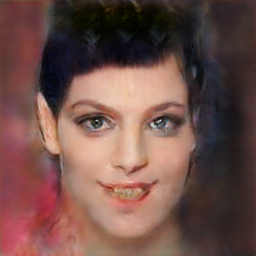

Supplement: Supplemental Information 4 [file peerj-cs-07-760-s004.zip › 02/206-targets-outputs.png]

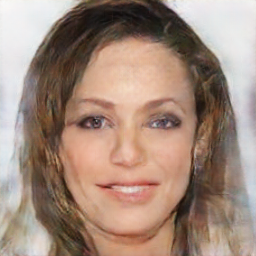

Supplement: Supplemental Information 4 [file peerj-cs-07-760-s004.zip › 02/207-targets-outputs.png]

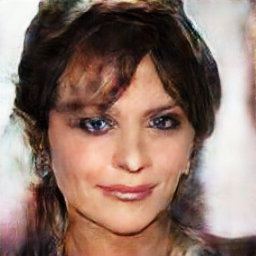

Supplement: Supplemental Information 4 [file peerj-cs-07-760-s004.zip › 02/208-targets-outputs.png]

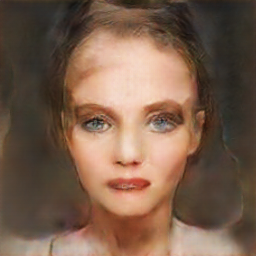

Supplement: Supplemental Information 4 [file peerj-cs-07-760-s004.zip › 02/209-targets-outputs.png]

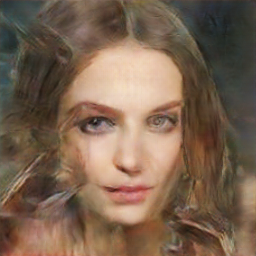

Supplement: Supplemental Information 4 [file peerj-cs-07-760-s004.zip › 02/210-targets-outputs.png]

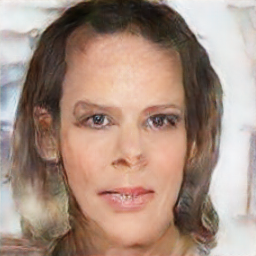

Supplement: Supplemental Information 4 [file peerj-cs-07-760-s004.zip › 02/211-targets-outputs.png]

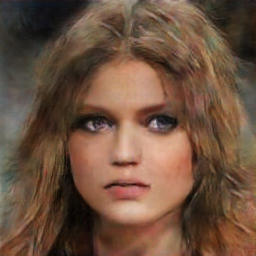

Supplement: Supplemental Information 4 [file peerj-cs-07-760-s004.zip › 02/212-targets-outputs.png]

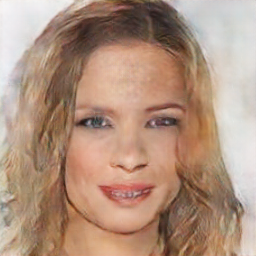

Supplement: Supplemental Information 4 [file peerj-cs-07-760-s004.zip › 02/213-targets-outputs.png]

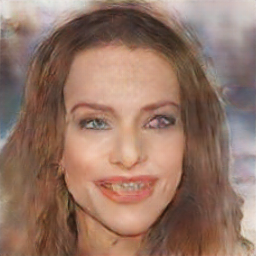

Supplement: Supplemental Information 4 [file peerj-cs-07-760-s004.zip › 02/214-targets-outputs.png]

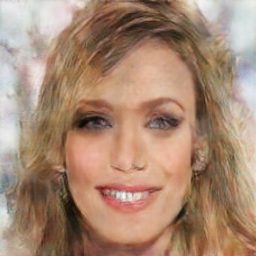

Supplement: Supplemental Information 4 [file peerj-cs-07-760-s004.zip › 02/215-targets-outputs.png]

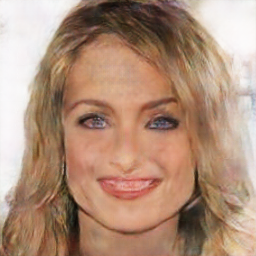

Supplement: Supplemental Information 4 [file peerj-cs-07-760-s004.zip › 02/216-targets-outputs.png]

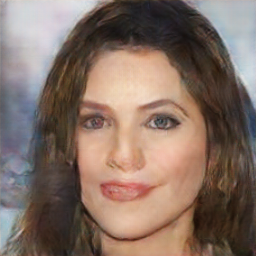

Supplement: Supplemental Information 4 [file peerj-cs-07-760-s004.zip › 02/217-targets-outputs.png]

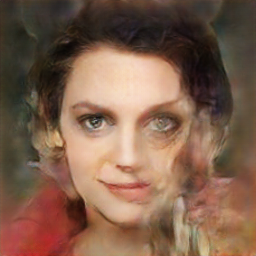

Supplement: Supplemental Information 4 [file peerj-cs-07-760-s004.zip › 02/218-targets-outputs.png]

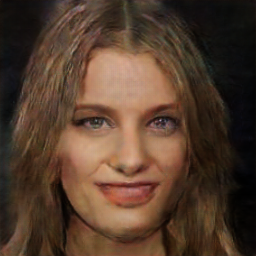

Supplement: Supplemental Information 4 [file peerj-cs-07-760-s004.zip › 02/219-targets-outputs.png]

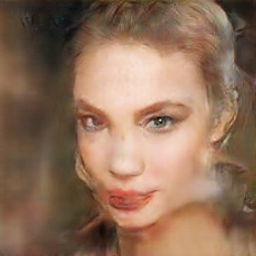

Supplement: Supplemental Information 4 [file peerj-cs-07-760-s004.zip › 02/220-targets-outputs.png]

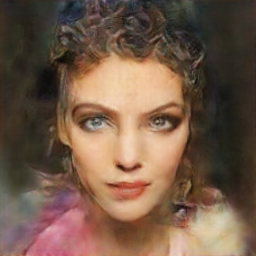

Supplement: Supplemental Information 4 [file peerj-cs-07-760-s004.zip › 02/221-targets-outputs.png]

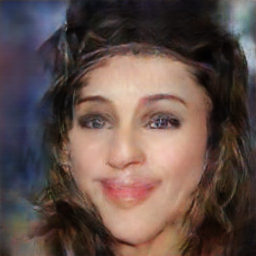

Supplement: Supplemental Information 4 [file peerj-cs-07-760-s004.zip › 02/222-targets-outputs.png]

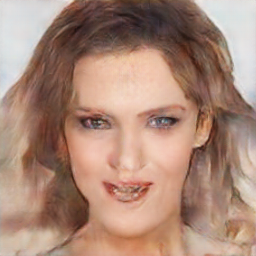

Supplement: Supplemental Information 4 [file peerj-cs-07-760-s004.zip › 02/223-targets-outputs.png]

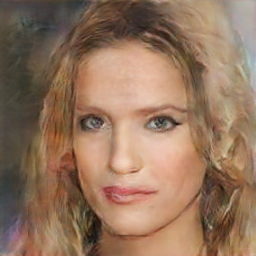

Supplement: Supplemental Information 4 [file peerj-cs-07-760-s004.zip › 02/224-targets-outputs.png]

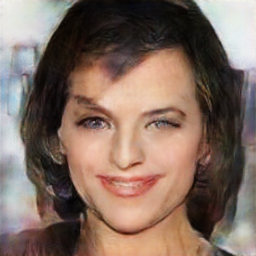

Supplement: Supplemental Information 4 [file peerj-cs-07-760-s004.zip › 02/225-targets-outputs.png]

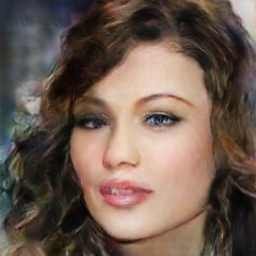

Supplement: Supplemental Information 4 [file peerj-cs-07-760-s004.zip › 02/226-targets-outputs.png]

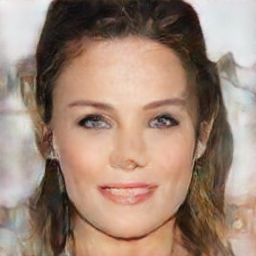

Supplement: Supplemental Information 4 [file peerj-cs-07-760-s004.zip › 02/227-targets-outputs.png]

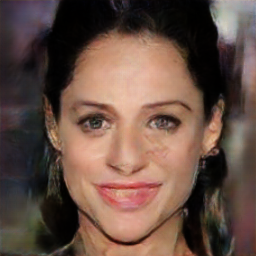

Supplement: Supplemental Information 4 [file peerj-cs-07-760-s004.zip › 02/228-targets-outputs.png]

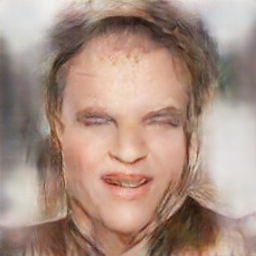

Supplement: Supplemental Information 4 [file peerj-cs-07-760-s004.zip › 02/229-targets-outputs.png]

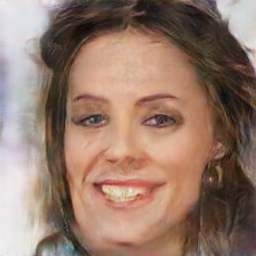

Supplement: Supplemental Information 4 [file peerj-cs-07-760-s004.zip › 02/230-targets-outputs.png]

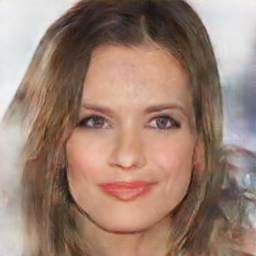

Supplement: Supplemental Information 4 [file peerj-cs-07-760-s004.zip › 02/231-targets-outputs.png]

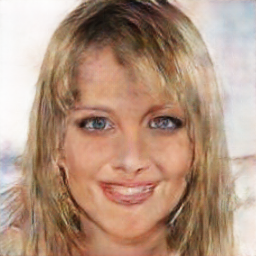

Supplement: Supplemental Information 4 [file peerj-cs-07-760-s004.zip › 02/232-targets-outputs.png]

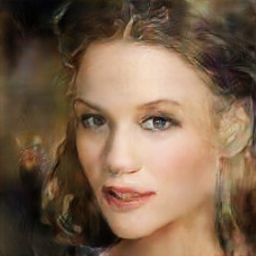

Supplement: Supplemental Information 4 [file peerj-cs-07-760-s004.zip › 02/233-targets-outputs.png]

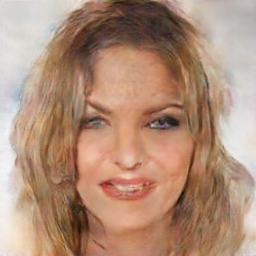

Supplement: Supplemental Information 4 [file peerj-cs-07-760-s004.zip › 02/234-targets-outputs.png]

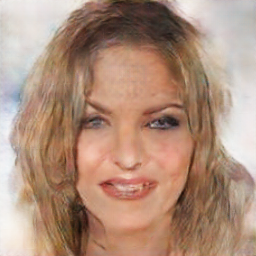

Supplement: Supplemental Information 4 [file peerj-cs-07-760-s004.zip › 02/235-targets-outputs.png]

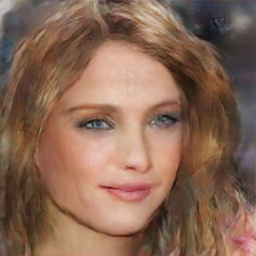

Supplement: Supplemental Information 4 [file peerj-cs-07-760-s004.zip › 02/236-targets-outputs.png]

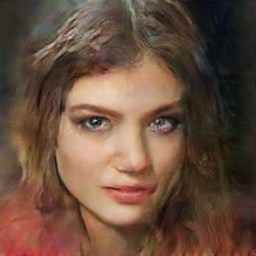

Supplement: Supplemental Information 4 [file peerj-cs-07-760-s004.zip › 02/237-targets-outputs.png]

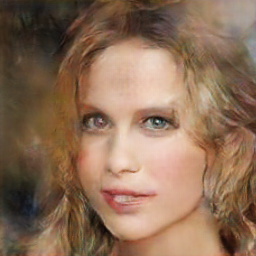

Supplement: Supplemental Information 4 [file peerj-cs-07-760-s004.zip › 02/238-targets-outputs.png]

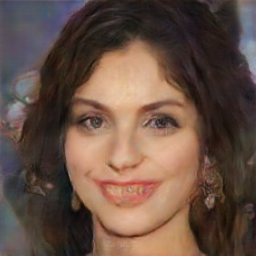

Supplement: Supplemental Information 4 [file peerj-cs-07-760-s004.zip › 02/239-targets-outputs.png]

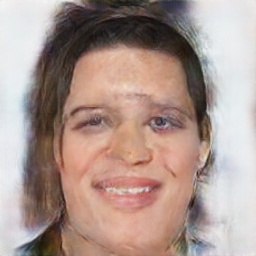

Supplement: Supplemental Information 4 [file peerj-cs-07-760-s004.zip › 02/240-targets-outputs.png]

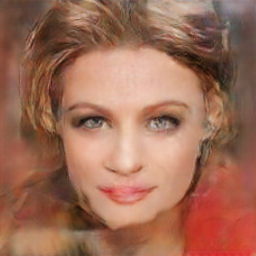

Supplement: Supplemental Information 4 [file peerj-cs-07-760-s004.zip › 02/241-targets-outputs.png]

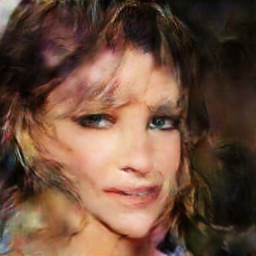

Supplement: Supplemental Information 4 [file peerj-cs-07-760-s004.zip › 02/242-targets-outputs.png]

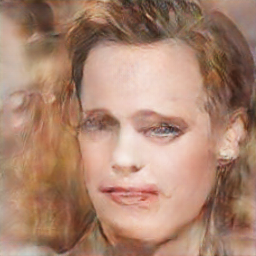

Supplement: Supplemental Information 4 [file peerj-cs-07-760-s004.zip › 02/243-targets-outputs.png]

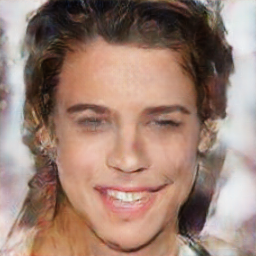

Supplement: Supplemental Information 4 [file peerj-cs-07-760-s004.zip › 02/244-targets-outputs.png]

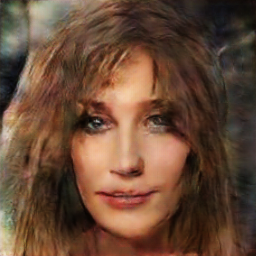

Supplement: Supplemental Information 4 [file peerj-cs-07-760-s004.zip › 02/245-targets-outputs.png]
