# Supplementary figures and images for: Small facial image dataset augmentation using conditional GANs based on incomplete edge feature input (part 2 of 6)
Source: PeerJ Comput Sci. 2021 Nov 17;7:e760. doi: 10.7717/peerj-cs.760 (PMC8627232; doi:10.7717/peerj-cs.760)

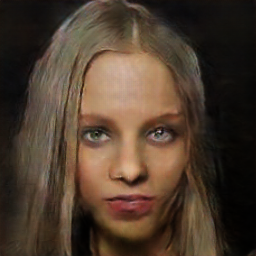

Supplement: Supplemental Information 3 [file peerj-cs-07-760-s003.zip › augmented facial images with sparse lines/single image blending/12-3-targets-outputs.png]

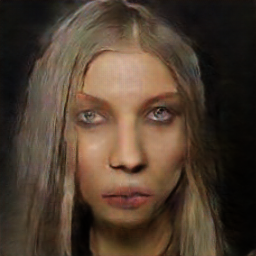

Supplement: Supplemental Information 3 [file peerj-cs-07-760-s003.zip › augmented facial images with sparse lines/single image blending/12-4-targets-outputs.png]

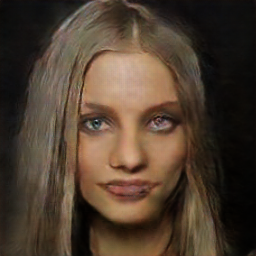

Supplement: Supplemental Information 3 [file peerj-cs-07-760-s003.zip › augmented facial images with sparse lines/single image blending/12-5-targets-outputs.png]

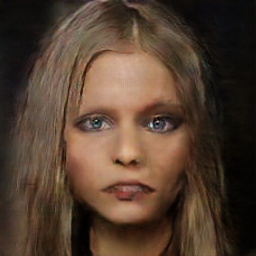

Supplement: Supplemental Information 3 [file peerj-cs-07-760-s003.zip › augmented facial images with sparse lines/single image blending/12-6-targets-outputs.png]

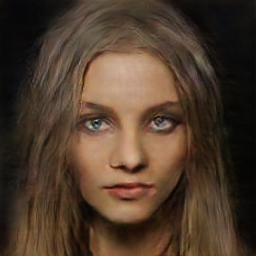

Supplement: Supplemental Information 3 [file peerj-cs-07-760-s003.zip › augmented facial images with sparse lines/single image blending/12-7-targets-outputs.png]

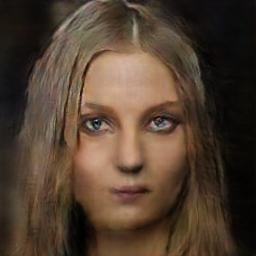

Supplement: Supplemental Information 3 [file peerj-cs-07-760-s003.zip › augmented facial images with sparse lines/single image blending/12-8-targets-outputs.png]

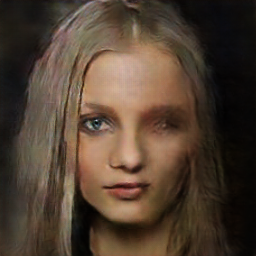

Supplement: Supplemental Information 3 [file peerj-cs-07-760-s003.zip › augmented facial images with sparse lines/single image blending/12-9-targets-outputs.png]

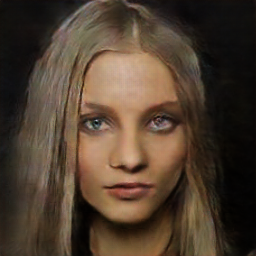

Supplement: Supplemental Information 3 [file peerj-cs-07-760-s003.zip › augmented facial images with sparse lines/single image blending/12-targets-outputs.png]

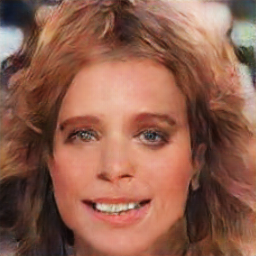

Supplement: Supplemental Information 3 [file peerj-cs-07-760-s003.zip › augmented facial images with sparse lines/single image blending/40-10-targets-outputs.png]

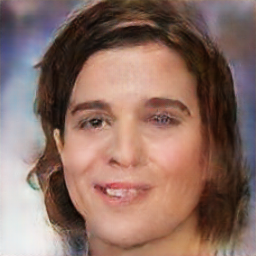

Supplement: Supplemental Information 3 [file peerj-cs-07-760-s003.zip › augmented facial images with sparse lines/single image blending/40-11-targets-outputs.png]

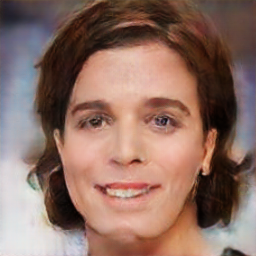

Supplement: Supplemental Information 3 [file peerj-cs-07-760-s003.zip › augmented facial images with sparse lines/single image blending/40-1-targets-outputs.png]

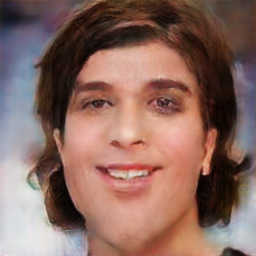

Supplement: Supplemental Information 3 [file peerj-cs-07-760-s003.zip › augmented facial images with sparse lines/single image blending/40-2-targets-outputs.png]

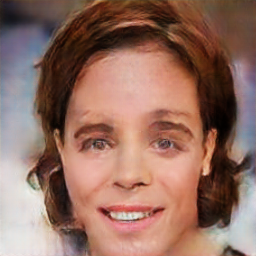

Supplement: Supplemental Information 3 [file peerj-cs-07-760-s003.zip › augmented facial images with sparse lines/single image blending/40-3-targets-outputs.png]

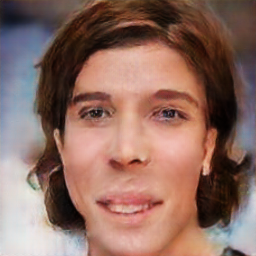

Supplement: Supplemental Information 3 [file peerj-cs-07-760-s003.zip › augmented facial images with sparse lines/single image blending/40-4-targets-outputs.png]

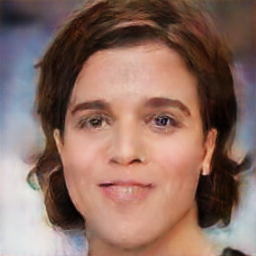

Supplement: Supplemental Information 3 [file peerj-cs-07-760-s003.zip › augmented facial images with sparse lines/single image blending/40-5-targets-outputs.png]

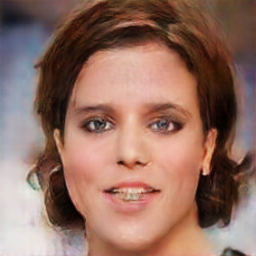

Supplement: Supplemental Information 3 [file peerj-cs-07-760-s003.zip › augmented facial images with sparse lines/single image blending/40-6-targets-outputs.png]

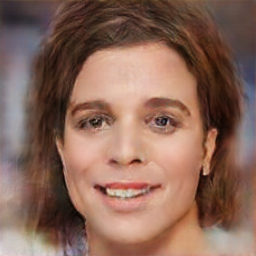

Supplement: Supplemental Information 3 [file peerj-cs-07-760-s003.zip › augmented facial images with sparse lines/single image blending/40-7-targets-outputs.png]

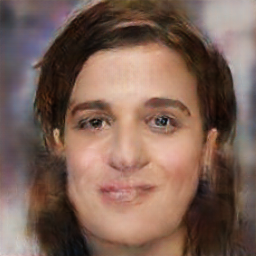

Supplement: Supplemental Information 3 [file peerj-cs-07-760-s003.zip › augmented facial images with sparse lines/single image blending/40-8-targets-outputs.png]

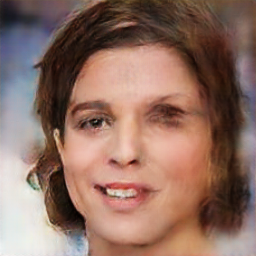

Supplement: Supplemental Information 3 [file peerj-cs-07-760-s003.zip › augmented facial images with sparse lines/single image blending/40-9-targets-outputs.png]

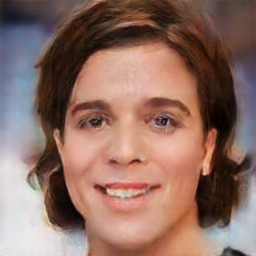

Supplement: Supplemental Information 3 [file peerj-cs-07-760-s003.zip › augmented facial images with sparse lines/single image blending/40-targets-outputs.png]

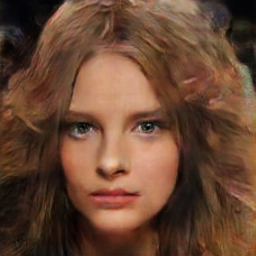

Supplement: Supplemental Information 3 [file peerj-cs-07-760-s003.zip › augmented facial images with sparse lines/single image blending/48-10-targets-outputs.png]

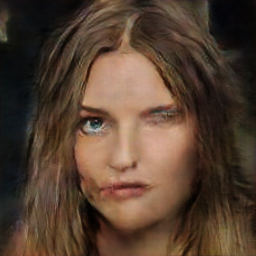

Supplement: Supplemental Information 3 [file peerj-cs-07-760-s003.zip › augmented facial images with sparse lines/single image blending/48-11-targets-outputs.png]

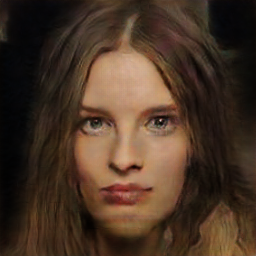

Supplement: Supplemental Information 3 [file peerj-cs-07-760-s003.zip › augmented facial images with sparse lines/single image blending/48-1-targets-outputs.png]

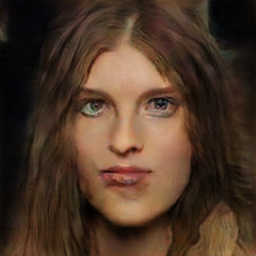

Supplement: Supplemental Information 3 [file peerj-cs-07-760-s003.zip › augmented facial images with sparse lines/single image blending/48-2-targets-outputs.png]

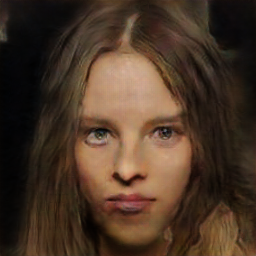

Supplement: Supplemental Information 3 [file peerj-cs-07-760-s003.zip › augmented facial images with sparse lines/single image blending/48-3-targets-outputs.png]

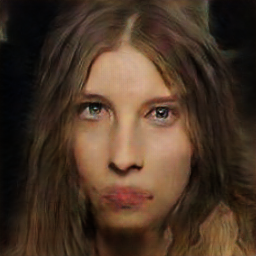

Supplement: Supplemental Information 3 [file peerj-cs-07-760-s003.zip › augmented facial images with sparse lines/single image blending/48-4-targets-outputs.png]

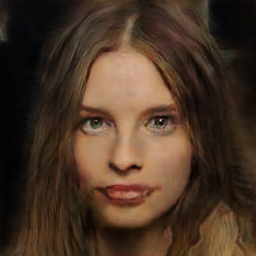

Supplement: Supplemental Information 3 [file peerj-cs-07-760-s003.zip › augmented facial images with sparse lines/single image blending/48-5-targets-outputs.png]

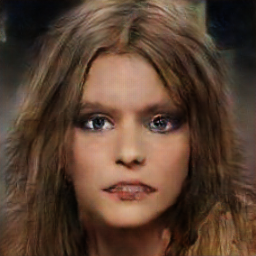

Supplement: Supplemental Information 3 [file peerj-cs-07-760-s003.zip › augmented facial images with sparse lines/single image blending/48-6-targets-outputs.png]

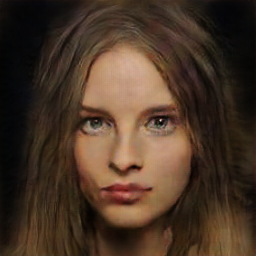

Supplement: Supplemental Information 3 [file peerj-cs-07-760-s003.zip › augmented facial images with sparse lines/single image blending/48-7-targets-outputs.png]

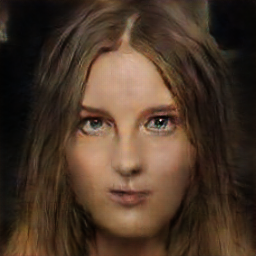

Supplement: Supplemental Information 3 [file peerj-cs-07-760-s003.zip › augmented facial images with sparse lines/single image blending/48-8-targets-outputs.png]

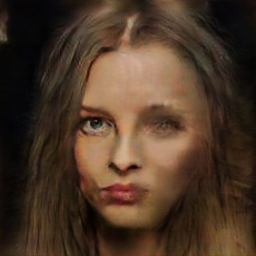

Supplement: Supplemental Information 3 [file peerj-cs-07-760-s003.zip › augmented facial images with sparse lines/single image blending/48-9-targets-outputs.png]

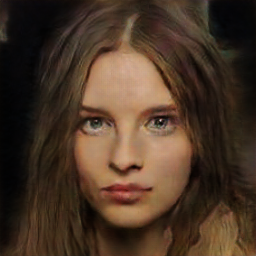

Supplement: Supplemental Information 3 [file peerj-cs-07-760-s003.zip › augmented facial images with sparse lines/single image blending/48-targets-outputs.png]

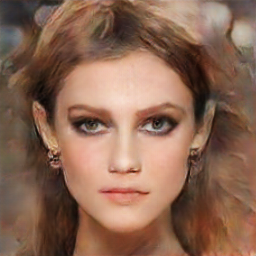

Supplement: Supplemental Information 3 [file peerj-cs-07-760-s003.zip › augmented facial images with sparse lines/single image blending/50-10-targets-outputs.png]

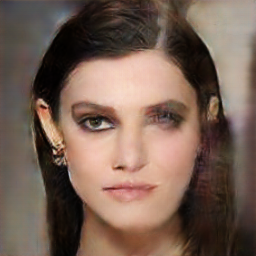

Supplement: Supplemental Information 3 [file peerj-cs-07-760-s003.zip › augmented facial images with sparse lines/single image blending/50-11-targets-outputs.png]

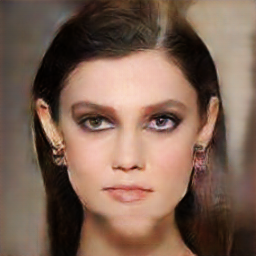

Supplement: Supplemental Information 3 [file peerj-cs-07-760-s003.zip › augmented facial images with sparse lines/single image blending/50-1-targets-outputs.png]

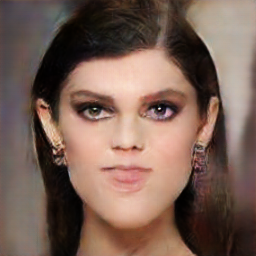

Supplement: Supplemental Information 3 [file peerj-cs-07-760-s003.zip › augmented facial images with sparse lines/single image blending/50-2-targets-outputs.png]

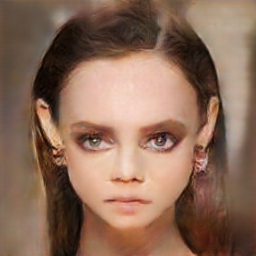

Supplement: Supplemental Information 3 [file peerj-cs-07-760-s003.zip › augmented facial images with sparse lines/single image blending/50-3-targets-outputs.png]

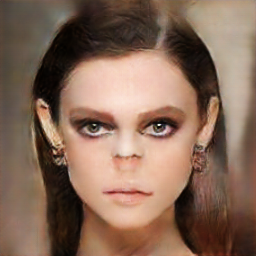

Supplement: Supplemental Information 3 [file peerj-cs-07-760-s003.zip › augmented facial images with sparse lines/single image blending/50-4-targets-outputs.png]

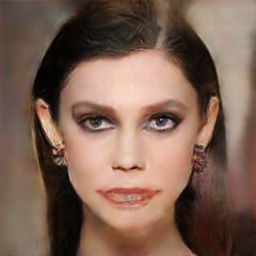

Supplement: Supplemental Information 3 [file peerj-cs-07-760-s003.zip › augmented facial images with sparse lines/single image blending/50-5-targets-outputs.png]

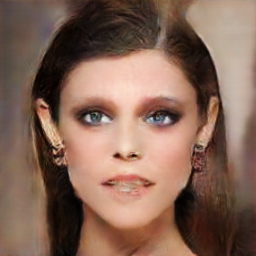

Supplement: Supplemental Information 3 [file peerj-cs-07-760-s003.zip › augmented facial images with sparse lines/single image blending/50-6-targets-outputs.png]

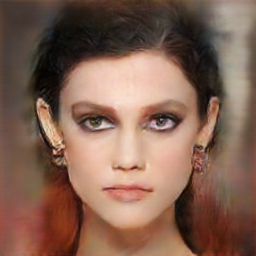

Supplement: Supplemental Information 3 [file peerj-cs-07-760-s003.zip › augmented facial images with sparse lines/single image blending/50-7-targets-outputs.png]

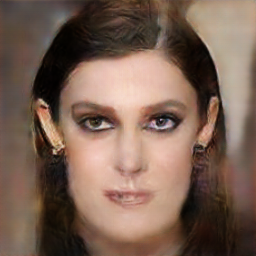

Supplement: Supplemental Information 3 [file peerj-cs-07-760-s003.zip › augmented facial images with sparse lines/single image blending/50-8-targets-outputs.png]

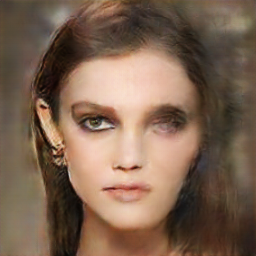

Supplement: Supplemental Information 3 [file peerj-cs-07-760-s003.zip › augmented facial images with sparse lines/single image blending/50-9-targets-outputs.png]

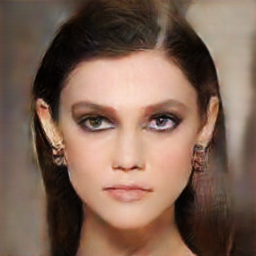

Supplement: Supplemental Information 3 [file peerj-cs-07-760-s003.zip › augmented facial images with sparse lines/single image blending/50-targets-outputs.png]

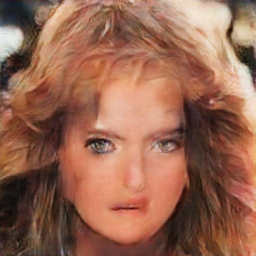

Supplement: Supplemental Information 3 [file peerj-cs-07-760-s003.zip › augmented facial images with sparse lines/single image blending/5-10-targets-outputs.png]

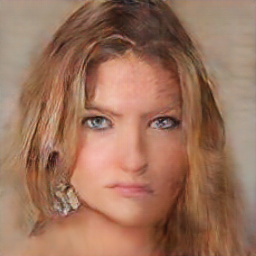

Supplement: Supplemental Information 3 [file peerj-cs-07-760-s003.zip › augmented facial images with sparse lines/single image blending/5-11-targets-outputs.png]

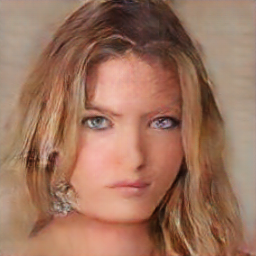

Supplement: Supplemental Information 3 [file peerj-cs-07-760-s003.zip › augmented facial images with sparse lines/single image blending/5-1-targets-outputs.png]

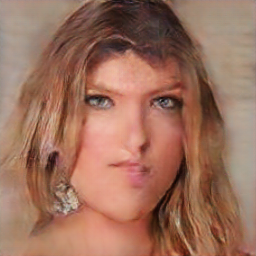

Supplement: Supplemental Information 3 [file peerj-cs-07-760-s003.zip › augmented facial images with sparse lines/single image blending/5-2-targets-outputs.png]

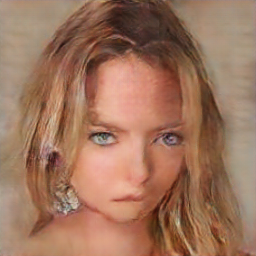

Supplement: Supplemental Information 3 [file peerj-cs-07-760-s003.zip › augmented facial images with sparse lines/single image blending/5-3-targets-outputs.png]

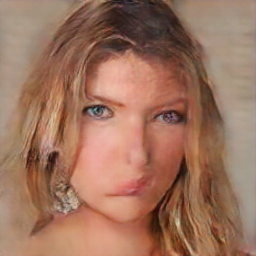

Supplement: Supplemental Information 3 [file peerj-cs-07-760-s003.zip › augmented facial images with sparse lines/single image blending/5-4-targets-outputs.png]

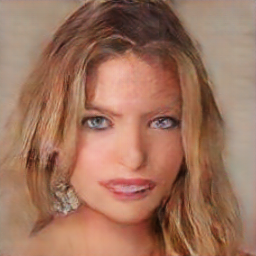

Supplement: Supplemental Information 3 [file peerj-cs-07-760-s003.zip › augmented facial images with sparse lines/single image blending/5-5-targets-outputs.png]

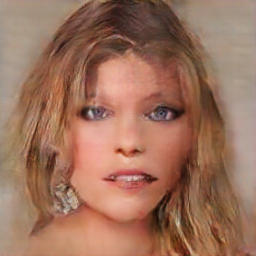

Supplement: Supplemental Information 3 [file peerj-cs-07-760-s003.zip › augmented facial images with sparse lines/single image blending/5-6-targets-outputs.png]

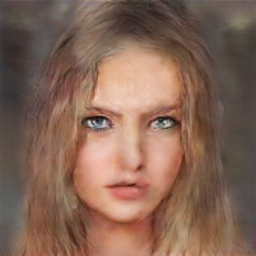

Supplement: Supplemental Information 3 [file peerj-cs-07-760-s003.zip › augmented facial images with sparse lines/single image blending/5-7-targets-outputs.png]

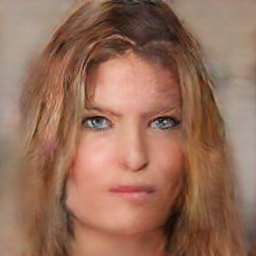

Supplement: Supplemental Information 3 [file peerj-cs-07-760-s003.zip › augmented facial images with sparse lines/single image blending/5-8-targets-outputs.png]

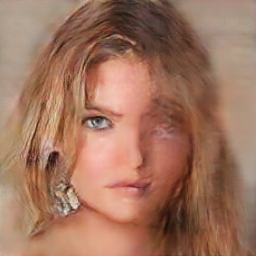

Supplement: Supplemental Information 3 [file peerj-cs-07-760-s003.zip › augmented facial images with sparse lines/single image blending/5-9-targets-outputs.png]

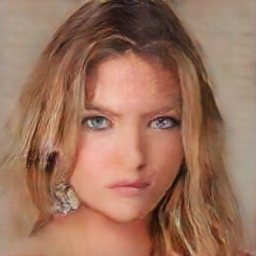

Supplement: Supplemental Information 3 [file peerj-cs-07-760-s003.zip › augmented facial images with sparse lines/single image blending/5-targets-outputs.png]

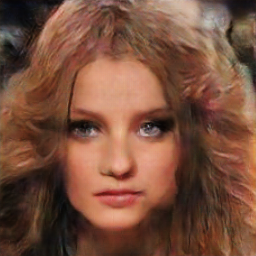

Supplement: Supplemental Information 3 [file peerj-cs-07-760-s003.zip › augmented facial images with sparse lines/two image swap/12-15-targets-outputs.png]

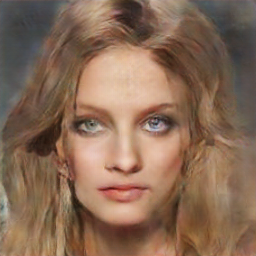

Supplement: Supplemental Information 3 [file peerj-cs-07-760-s003.zip › augmented facial images with sparse lines/two image swap/12-20-targets-outputs.png]

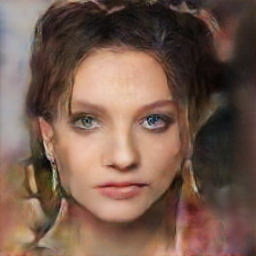

Supplement: Supplemental Information 3 [file peerj-cs-07-760-s003.zip › augmented facial images with sparse lines/two image swap/12-27-targets-outputs.png]

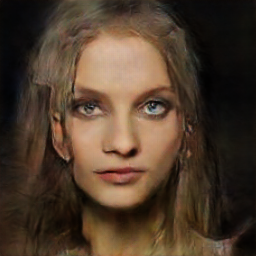

Supplement: Supplemental Information 3 [file peerj-cs-07-760-s003.zip › augmented facial images with sparse lines/two image swap/12-42-targets-outputs.png]

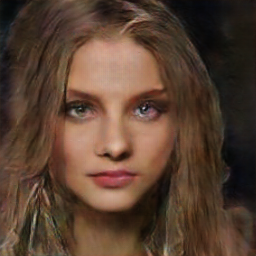

Supplement: Supplemental Information 3 [file peerj-cs-07-760-s003.zip › augmented facial images with sparse lines/two image swap/12-46-targets-outputs.png]

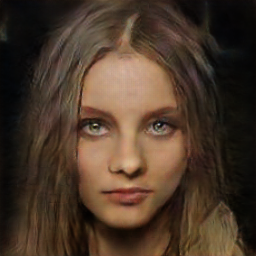

Supplement: Supplemental Information 3 [file peerj-cs-07-760-s003.zip › augmented facial images with sparse lines/two image swap/12-48-targets-outputs.png]

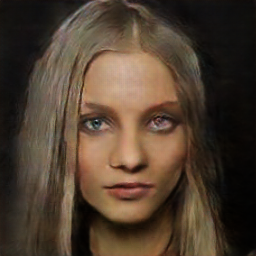

Supplement: Supplemental Information 3 [file peerj-cs-07-760-s003.zip › augmented facial images with sparse lines/two image swap/12-targets-outputs.png]

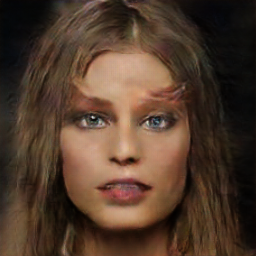

Supplement: Supplemental Information 3 [file peerj-cs-07-760-s003.zip › augmented facial images with sparse lines/two image swap/15-12-targets-outputs.png]

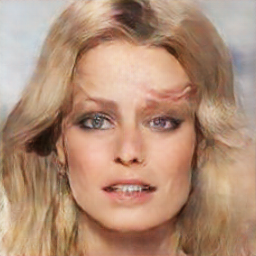

Supplement: Supplemental Information 3 [file peerj-cs-07-760-s003.zip › augmented facial images with sparse lines/two image swap/15-20-targets-outputs.png]

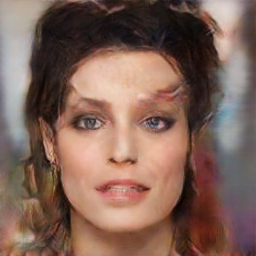

Supplement: Supplemental Information 3 [file peerj-cs-07-760-s003.zip › augmented facial images with sparse lines/two image swap/15-27-targets-outputs.png]

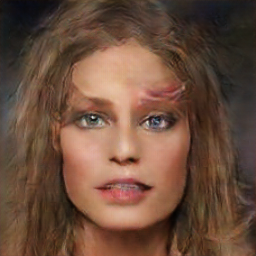

Supplement: Supplemental Information 3 [file peerj-cs-07-760-s003.zip › augmented facial images with sparse lines/two image swap/15-42-targets-outputs.png]

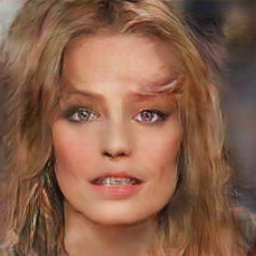

Supplement: Supplemental Information 3 [file peerj-cs-07-760-s003.zip › augmented facial images with sparse lines/two image swap/15-46-targets-outputs.png]

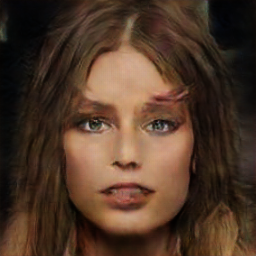

Supplement: Supplemental Information 3 [file peerj-cs-07-760-s003.zip › augmented facial images with sparse lines/two image swap/15-48-targets-outputs.png]

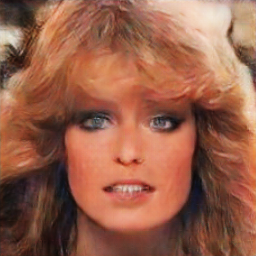

Supplement: Supplemental Information 3 [file peerj-cs-07-760-s003.zip › augmented facial images with sparse lines/two image swap/15-targets-outputs.png]

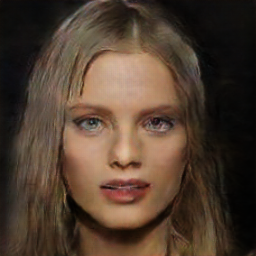

Supplement: Supplemental Information 3 [file peerj-cs-07-760-s003.zip › augmented facial images with sparse lines/two image swap/20-12-targets-outputs.png]

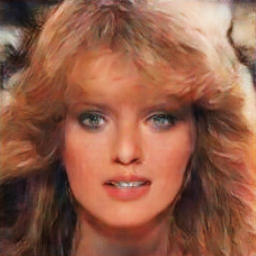

Supplement: Supplemental Information 3 [file peerj-cs-07-760-s003.zip › augmented facial images with sparse lines/two image swap/20-15-targets-outputs.png]

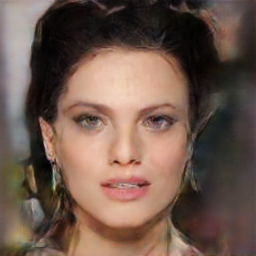

Supplement: Supplemental Information 3 [file peerj-cs-07-760-s003.zip › augmented facial images with sparse lines/two image swap/20-27-targets-outputs.png]

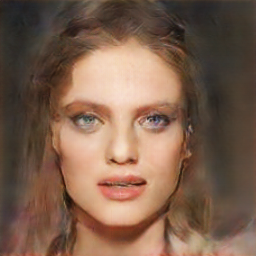

Supplement: Supplemental Information 3 [file peerj-cs-07-760-s003.zip › augmented facial images with sparse lines/two image swap/20-42-targets-outputs.png]

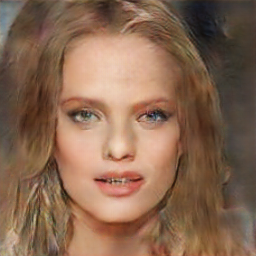

Supplement: Supplemental Information 3 [file peerj-cs-07-760-s003.zip › augmented facial images with sparse lines/two image swap/20-46-targets-outputs.png]

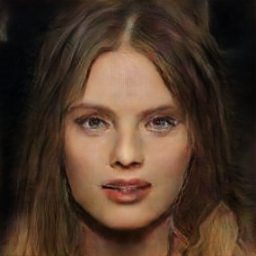

Supplement: Supplemental Information 3 [file peerj-cs-07-760-s003.zip › augmented facial images with sparse lines/two image swap/20-48-targets-outputs.png]

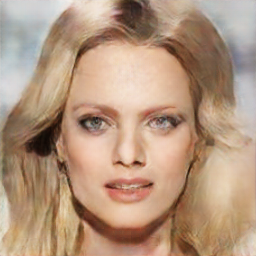

Supplement: Supplemental Information 3 [file peerj-cs-07-760-s003.zip › augmented facial images with sparse lines/two image swap/20-targets-outputs.png]

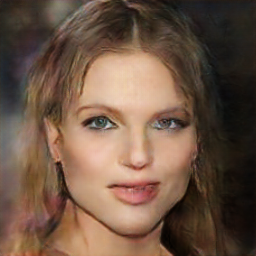

Supplement: Supplemental Information 3 [file peerj-cs-07-760-s003.zip › augmented facial images with sparse lines/two image swap/27-12-targets-outputs.png]

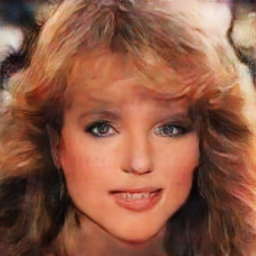

Supplement: Supplemental Information 3 [file peerj-cs-07-760-s003.zip › augmented facial images with sparse lines/two image swap/27-15-targets-outputs.png]

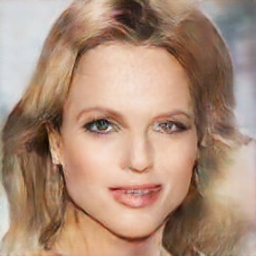

Supplement: Supplemental Information 3 [file peerj-cs-07-760-s003.zip › augmented facial images with sparse lines/two image swap/27-20-targets-outputs.png]

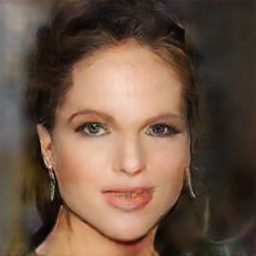

Supplement: Supplemental Information 3 [file peerj-cs-07-760-s003.zip › augmented facial images with sparse lines/two image swap/27-42-targets-outputs.png]

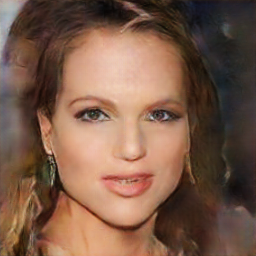

Supplement: Supplemental Information 3 [file peerj-cs-07-760-s003.zip › augmented facial images with sparse lines/two image swap/27-46-targets-outputs.png]

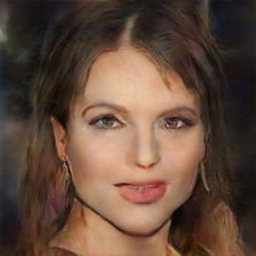

Supplement: Supplemental Information 3 [file peerj-cs-07-760-s003.zip › augmented facial images with sparse lines/two image swap/27-48-targets-outputs.png]

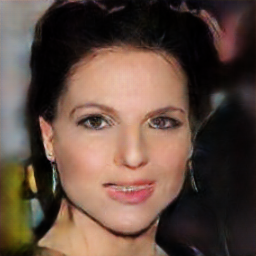

Supplement: Supplemental Information 3 [file peerj-cs-07-760-s003.zip › augmented facial images with sparse lines/two image swap/27-targets-outputs.png]

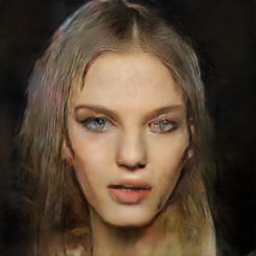

Supplement: Supplemental Information 3 [file peerj-cs-07-760-s003.zip › augmented facial images with sparse lines/two image swap/42-12-targets-outputs.png]

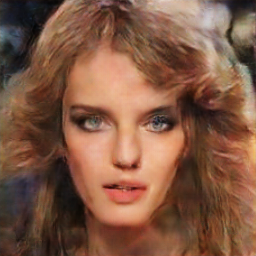

Supplement: Supplemental Information 3 [file peerj-cs-07-760-s003.zip › augmented facial images with sparse lines/two image swap/42-15-targets-outputs.png]

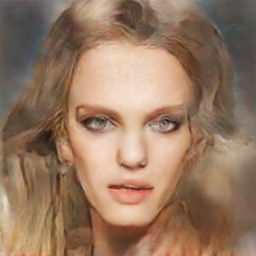

Supplement: Supplemental Information 3 [file peerj-cs-07-760-s003.zip › augmented facial images with sparse lines/two image swap/42-20-targets-outputs.png]

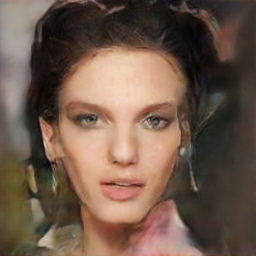

Supplement: Supplemental Information 3 [file peerj-cs-07-760-s003.zip › augmented facial images with sparse lines/two image swap/42-27-targets-outputs.png]

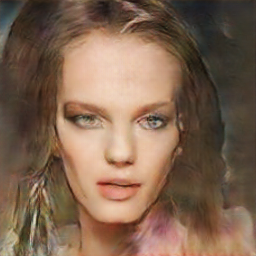

Supplement: Supplemental Information 3 [file peerj-cs-07-760-s003.zip › augmented facial images with sparse lines/two image swap/42-46-targets-outputs.png]

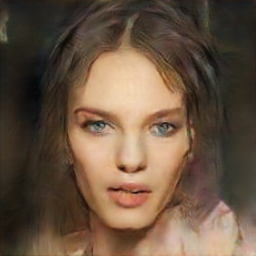

Supplement: Supplemental Information 3 [file peerj-cs-07-760-s003.zip › augmented facial images with sparse lines/two image swap/42-48-targets-outputs.png]

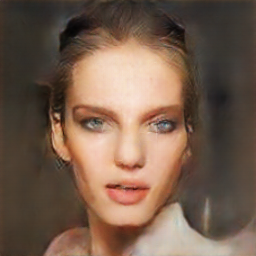

Supplement: Supplemental Information 3 [file peerj-cs-07-760-s003.zip › augmented facial images with sparse lines/two image swap/42-targets-outputs.png]

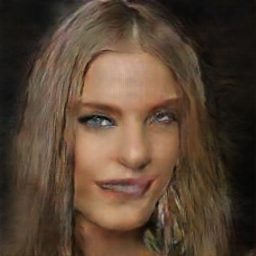

Supplement: Supplemental Information 3 [file peerj-cs-07-760-s003.zip › augmented facial images with sparse lines/two image swap/46-12-targets-outputs.png]

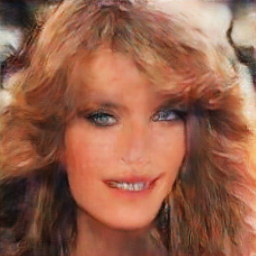

Supplement: Supplemental Information 3 [file peerj-cs-07-760-s003.zip › augmented facial images with sparse lines/two image swap/46-15-targets-outputs.png]

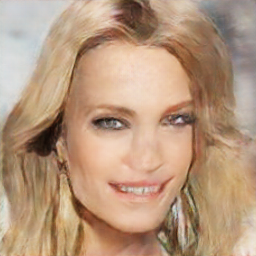

Supplement: Supplemental Information 3 [file peerj-cs-07-760-s003.zip › augmented facial images with sparse lines/two image swap/46-20-targets-outputs.png]

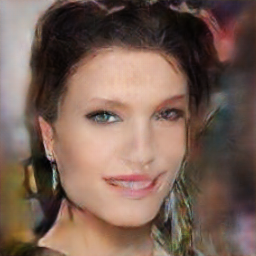

Supplement: Supplemental Information 3 [file peerj-cs-07-760-s003.zip › augmented facial images with sparse lines/two image swap/46-27-targets-outputs.png]

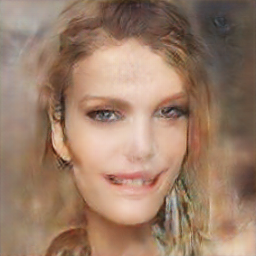

Supplement: Supplemental Information 3 [file peerj-cs-07-760-s003.zip › augmented facial images with sparse lines/two image swap/46-42-targets-outputs.png]

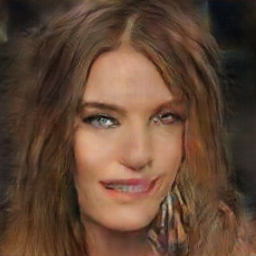

Supplement: Supplemental Information 3 [file peerj-cs-07-760-s003.zip › augmented facial images with sparse lines/two image swap/46-48-targets-outputs.png]

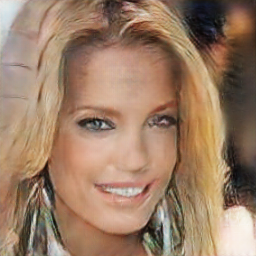

Supplement: Supplemental Information 3 [file peerj-cs-07-760-s003.zip › augmented facial images with sparse lines/two image swap/46-targets-outputs.png]

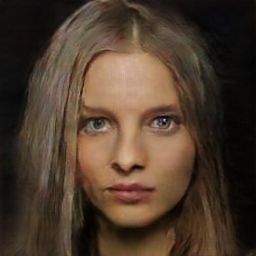

Supplement: Supplemental Information 3 [file peerj-cs-07-760-s003.zip › augmented facial images with sparse lines/two image swap/48-12-targets-outputs.png]

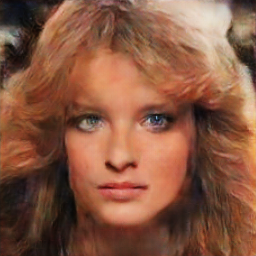

Supplement: Supplemental Information 3 [file peerj-cs-07-760-s003.zip › augmented facial images with sparse lines/two image swap/48-15-targets-outputs.png]
