# Supplementary figures and images for: Small facial image dataset augmentation using conditional GANs based on incomplete edge feature input (part 4 of 6)
Source: PeerJ Comput Sci. 2021 Nov 17;7:e760. doi: 10.7717/peerj-cs.760 (PMC8627232; doi:10.7717/peerj-cs.760)

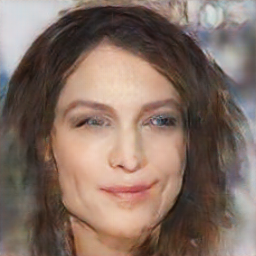

Supplement: Supplemental Information 4 [file peerj-cs-07-760-s004.zip › 02/246-targets-outputs.png]

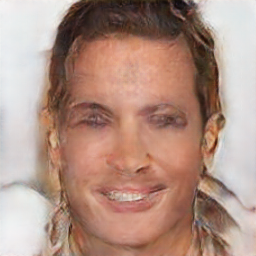

Supplement: Supplemental Information 4 [file peerj-cs-07-760-s004.zip › 02/247-targets-outputs.png]

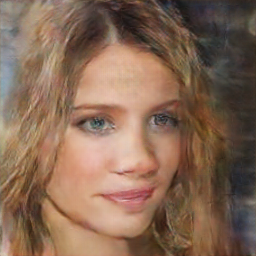

Supplement: Supplemental Information 4 [file peerj-cs-07-760-s004.zip › 02/248-targets-outputs.png]

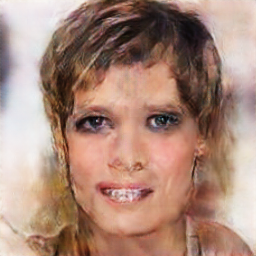

Supplement: Supplemental Information 4 [file peerj-cs-07-760-s004.zip › 02/249-targets-outputs.png]

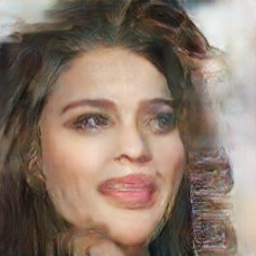

Supplement: Supplemental Information 4 [file peerj-cs-07-760-s004.zip › 02/250-targets-outputs.png]

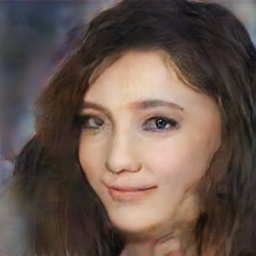

Supplement: Supplemental Information 4 [file peerj-cs-07-760-s004.zip › 03/201-targets-outputs.png]

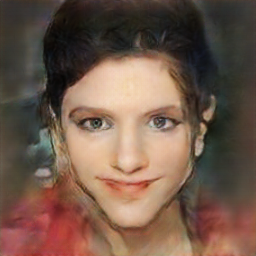

Supplement: Supplemental Information 4 [file peerj-cs-07-760-s004.zip › 03/202-targets-outputs.png]

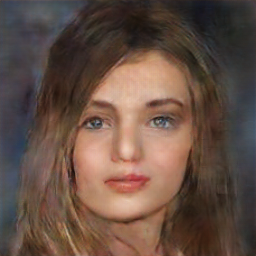

Supplement: Supplemental Information 4 [file peerj-cs-07-760-s004.zip › 03/203-targets-outputs.png]

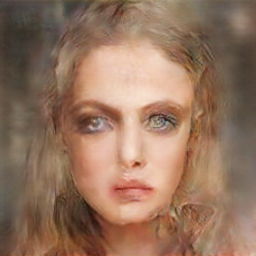

Supplement: Supplemental Information 4 [file peerj-cs-07-760-s004.zip › 03/204-targets-outputs.png]

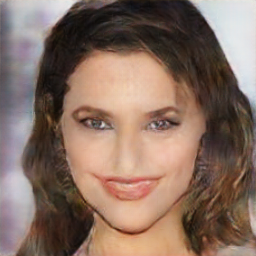

Supplement: Supplemental Information 4 [file peerj-cs-07-760-s004.zip › 03/205-targets-outputs.png]

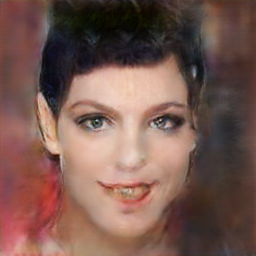

Supplement: Supplemental Information 4 [file peerj-cs-07-760-s004.zip › 03/206-targets-outputs.png]

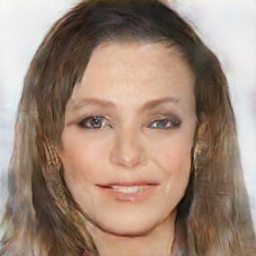

Supplement: Supplemental Information 4 [file peerj-cs-07-760-s004.zip › 03/207-targets-outputs.png]

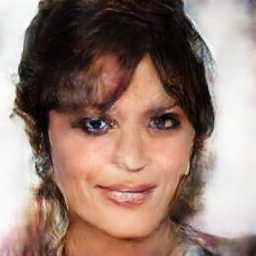

Supplement: Supplemental Information 4 [file peerj-cs-07-760-s004.zip › 03/208-targets-outputs.png]

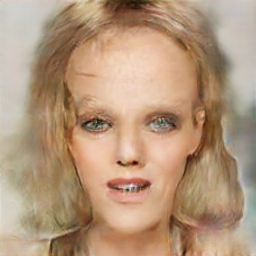

Supplement: Supplemental Information 4 [file peerj-cs-07-760-s004.zip › 03/209-targets-outputs.png]

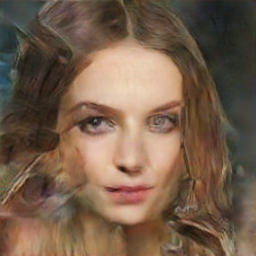

Supplement: Supplemental Information 4 [file peerj-cs-07-760-s004.zip › 03/210-targets-outputs.png]

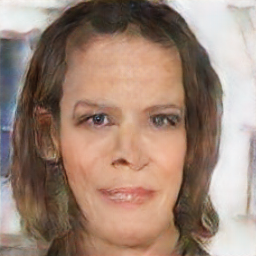

Supplement: Supplemental Information 4 [file peerj-cs-07-760-s004.zip › 03/211-targets-outputs.png]

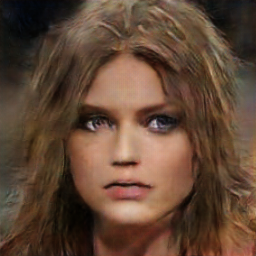

Supplement: Supplemental Information 4 [file peerj-cs-07-760-s004.zip › 03/212-targets-outputs.png]

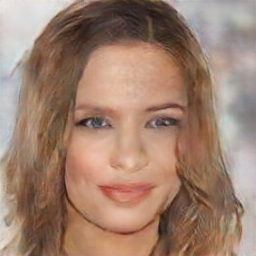

Supplement: Supplemental Information 4 [file peerj-cs-07-760-s004.zip › 03/213-targets-outputs.png]

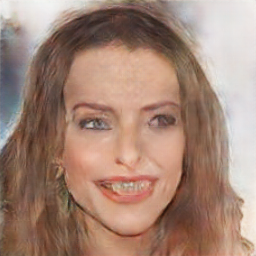

Supplement: Supplemental Information 4 [file peerj-cs-07-760-s004.zip › 03/214-targets-outputs.png]

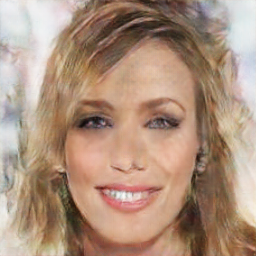

Supplement: Supplemental Information 4 [file peerj-cs-07-760-s004.zip › 03/215-targets-outputs.png]

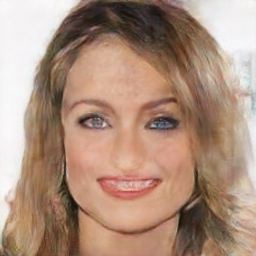

Supplement: Supplemental Information 4 [file peerj-cs-07-760-s004.zip › 03/216-targets-outputs.png]

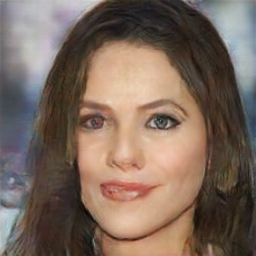

Supplement: Supplemental Information 4 [file peerj-cs-07-760-s004.zip › 03/217-targets-outputs.png]

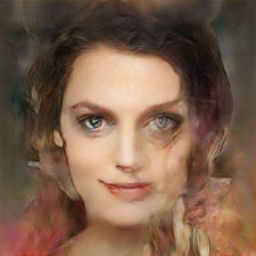

Supplement: Supplemental Information 4 [file peerj-cs-07-760-s004.zip › 03/218-targets-outputs.png]

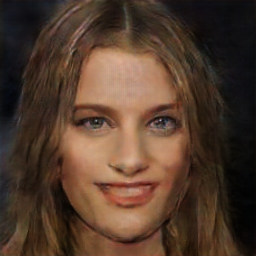

Supplement: Supplemental Information 4 [file peerj-cs-07-760-s004.zip › 03/219-targets-outputs.png]

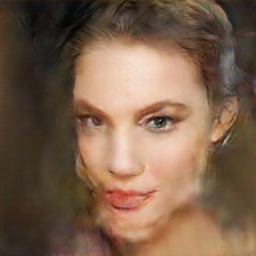

Supplement: Supplemental Information 4 [file peerj-cs-07-760-s004.zip › 03/220-targets-outputs.png]

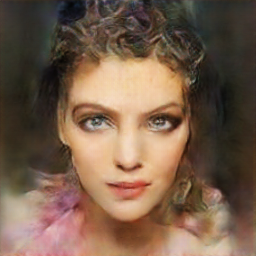

Supplement: Supplemental Information 4 [file peerj-cs-07-760-s004.zip › 03/221-targets-outputs.png]

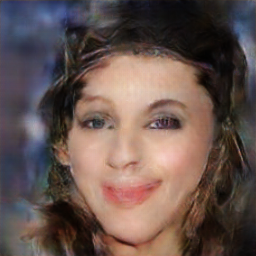

Supplement: Supplemental Information 4 [file peerj-cs-07-760-s004.zip › 03/222-targets-outputs.png]

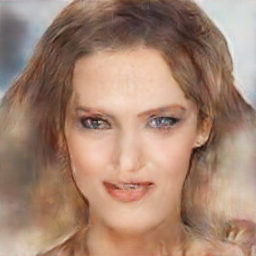

Supplement: Supplemental Information 4 [file peerj-cs-07-760-s004.zip › 03/223-targets-outputs.png]

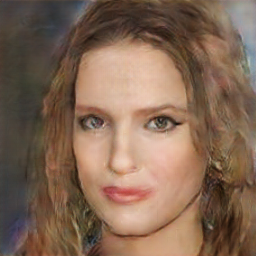

Supplement: Supplemental Information 4 [file peerj-cs-07-760-s004.zip › 03/224-targets-outputs.png]

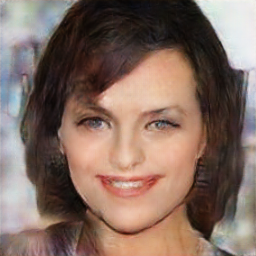

Supplement: Supplemental Information 4 [file peerj-cs-07-760-s004.zip › 03/225-targets-outputs.png]

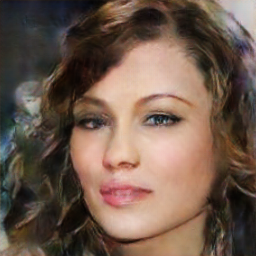

Supplement: Supplemental Information 4 [file peerj-cs-07-760-s004.zip › 03/226-targets-outputs.png]

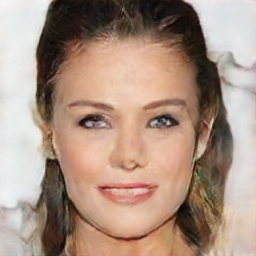

Supplement: Supplemental Information 4 [file peerj-cs-07-760-s004.zip › 03/227-targets-outputs.png]

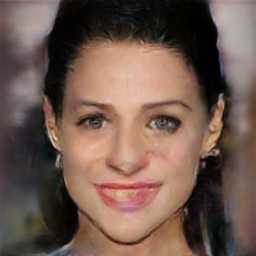

Supplement: Supplemental Information 4 [file peerj-cs-07-760-s004.zip › 03/228-targets-outputs.png]

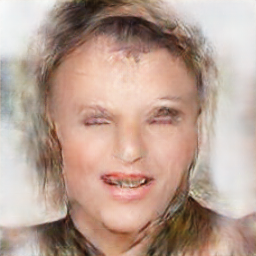

Supplement: Supplemental Information 4 [file peerj-cs-07-760-s004.zip › 03/229-targets-outputs.png]

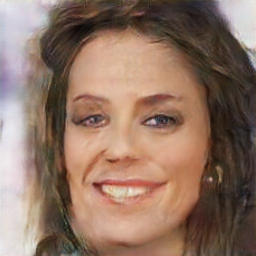

Supplement: Supplemental Information 4 [file peerj-cs-07-760-s004.zip › 03/230-targets-outputs.png]

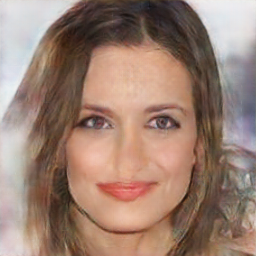

Supplement: Supplemental Information 4 [file peerj-cs-07-760-s004.zip › 03/231-targets-outputs.png]

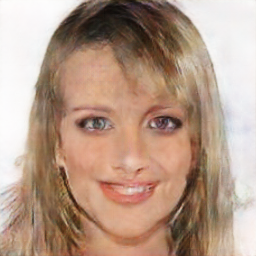

Supplement: Supplemental Information 4 [file peerj-cs-07-760-s004.zip › 03/232-targets-outputs.png]

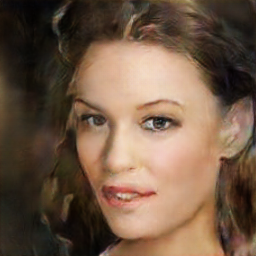

Supplement: Supplemental Information 4 [file peerj-cs-07-760-s004.zip › 03/233-targets-outputs.png]

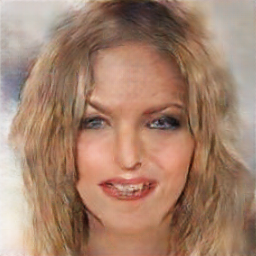

Supplement: Supplemental Information 4 [file peerj-cs-07-760-s004.zip › 03/234-targets-outputs.png]

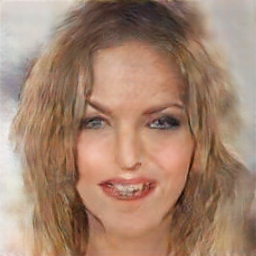

Supplement: Supplemental Information 4 [file peerj-cs-07-760-s004.zip › 03/235-targets-outputs.png]

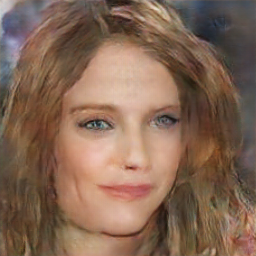

Supplement: Supplemental Information 4 [file peerj-cs-07-760-s004.zip › 03/236-targets-outputs.png]

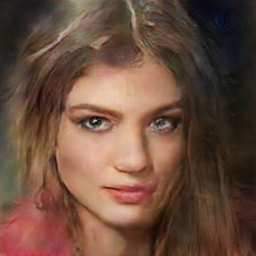

Supplement: Supplemental Information 4 [file peerj-cs-07-760-s004.zip › 03/237-targets-outputs.png]

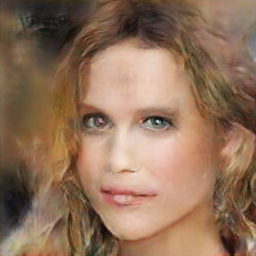

Supplement: Supplemental Information 4 [file peerj-cs-07-760-s004.zip › 03/238-targets-outputs.png]

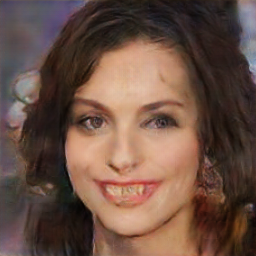

Supplement: Supplemental Information 4 [file peerj-cs-07-760-s004.zip › 03/239-targets-outputs.png]

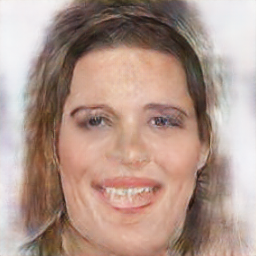

Supplement: Supplemental Information 4 [file peerj-cs-07-760-s004.zip › 03/240-targets-outputs.png]

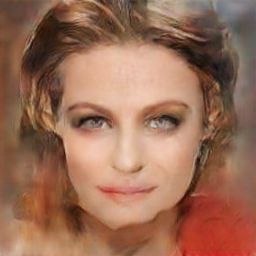

Supplement: Supplemental Information 4 [file peerj-cs-07-760-s004.zip › 03/241-targets-outputs.png]

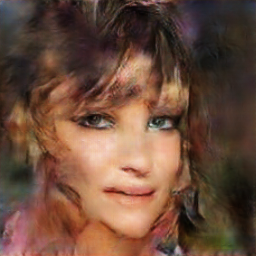

Supplement: Supplemental Information 4 [file peerj-cs-07-760-s004.zip › 03/242-targets-outputs.png]

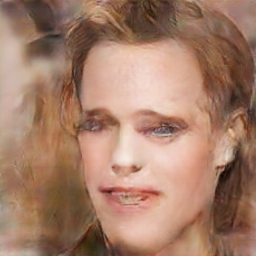

Supplement: Supplemental Information 4 [file peerj-cs-07-760-s004.zip › 03/243-targets-outputs.png]

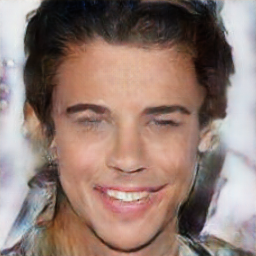

Supplement: Supplemental Information 4 [file peerj-cs-07-760-s004.zip › 03/244-targets-outputs.png]

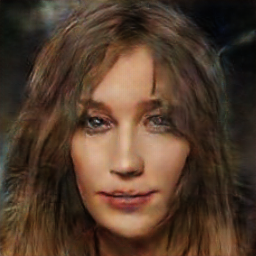

Supplement: Supplemental Information 4 [file peerj-cs-07-760-s004.zip › 03/245-targets-outputs.png]

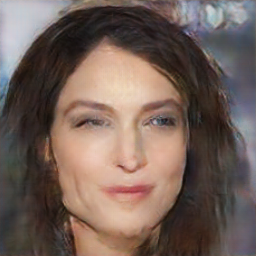

Supplement: Supplemental Information 4 [file peerj-cs-07-760-s004.zip › 03/246-targets-outputs.png]

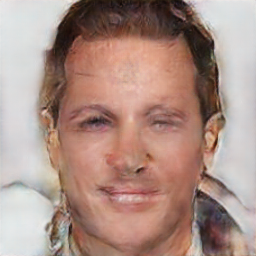

Supplement: Supplemental Information 4 [file peerj-cs-07-760-s004.zip › 03/247-targets-outputs.png]

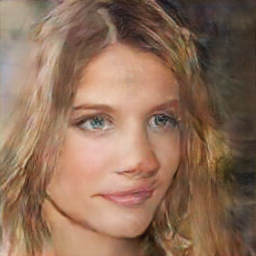

Supplement: Supplemental Information 4 [file peerj-cs-07-760-s004.zip › 03/248-targets-outputs.png]

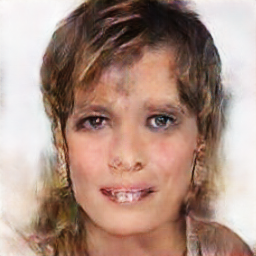

Supplement: Supplemental Information 4 [file peerj-cs-07-760-s004.zip › 03/249-targets-outputs.png]

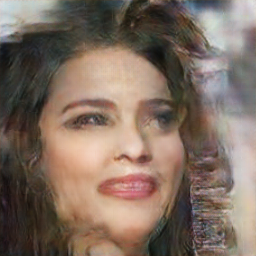

Supplement: Supplemental Information 4 [file peerj-cs-07-760-s004.zip › 03/250-targets-outputs.png]

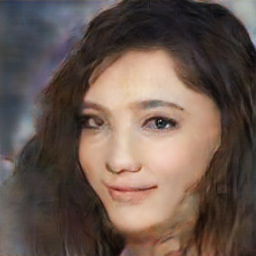

Supplement: Supplemental Information 5 [file peerj-cs-07-760-s005.zip › 04/201-targets-outputs.png]

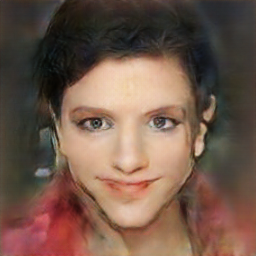

Supplement: Supplemental Information 5 [file peerj-cs-07-760-s005.zip › 04/202-targets-outputs.png]

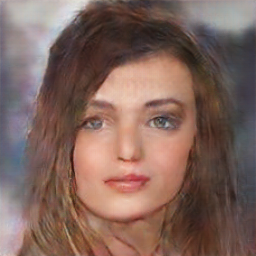

Supplement: Supplemental Information 5 [file peerj-cs-07-760-s005.zip › 04/203-targets-outputs.png]

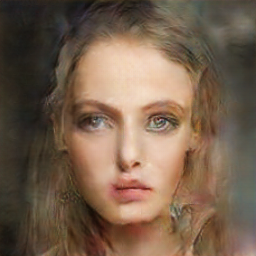

Supplement: Supplemental Information 5 [file peerj-cs-07-760-s005.zip › 04/204-targets-outputs.png]

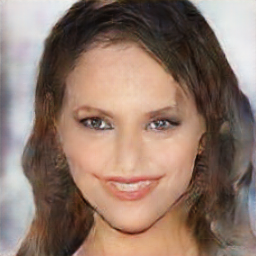

Supplement: Supplemental Information 5 [file peerj-cs-07-760-s005.zip › 04/205-targets-outputs.png]

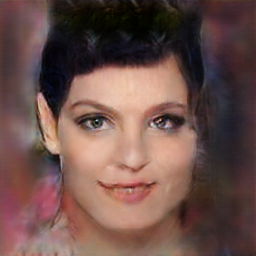

Supplement: Supplemental Information 5 [file peerj-cs-07-760-s005.zip › 04/206-targets-outputs.png]

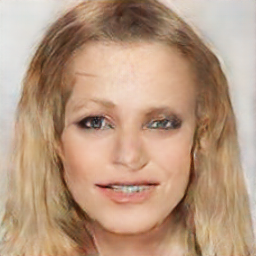

Supplement: Supplemental Information 5 [file peerj-cs-07-760-s005.zip › 04/207-targets-outputs.png]

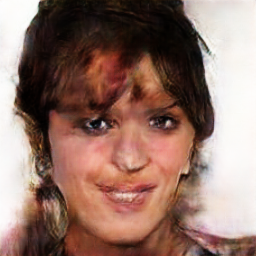

Supplement: Supplemental Information 5 [file peerj-cs-07-760-s005.zip › 04/208-targets-outputs.png]

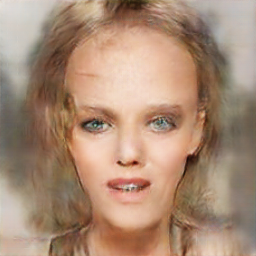

Supplement: Supplemental Information 5 [file peerj-cs-07-760-s005.zip › 04/209-targets-outputs.png]

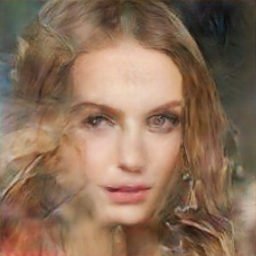

Supplement: Supplemental Information 5 [file peerj-cs-07-760-s005.zip › 04/210-targets-outputs.png]

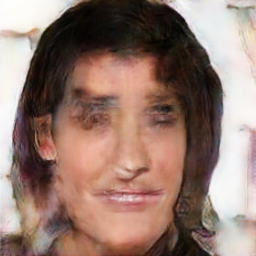

Supplement: Supplemental Information 5 [file peerj-cs-07-760-s005.zip › 04/211-targets-outputs.png]

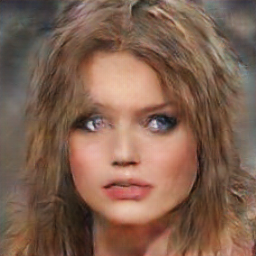

Supplement: Supplemental Information 5 [file peerj-cs-07-760-s005.zip › 04/212-targets-outputs.png]

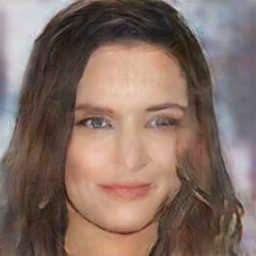

Supplement: Supplemental Information 5 [file peerj-cs-07-760-s005.zip › 04/213-targets-outputs.png]

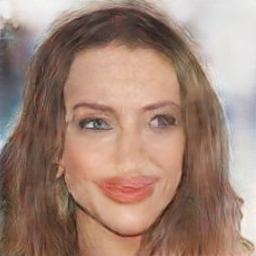

Supplement: Supplemental Information 5 [file peerj-cs-07-760-s005.zip › 04/214-targets-outputs.png]

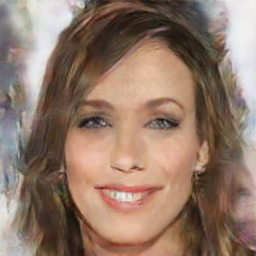

Supplement: Supplemental Information 5 [file peerj-cs-07-760-s005.zip › 04/215-targets-outputs.png]

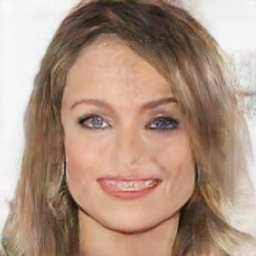

Supplement: Supplemental Information 5 [file peerj-cs-07-760-s005.zip › 04/216-targets-outputs.png]

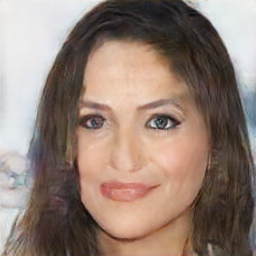

Supplement: Supplemental Information 5 [file peerj-cs-07-760-s005.zip › 04/217-targets-outputs.png]

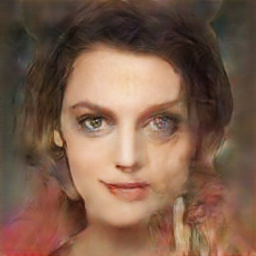

Supplement: Supplemental Information 5 [file peerj-cs-07-760-s005.zip › 04/218-targets-outputs.png]

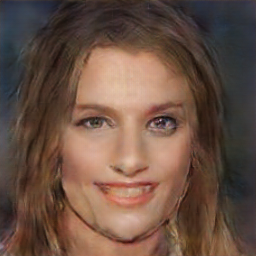

Supplement: Supplemental Information 5 [file peerj-cs-07-760-s005.zip › 04/219-targets-outputs.png]

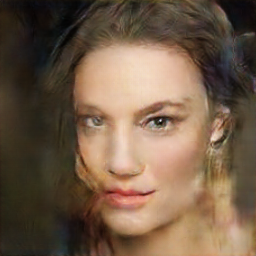

Supplement: Supplemental Information 5 [file peerj-cs-07-760-s005.zip › 04/220-targets-outputs.png]

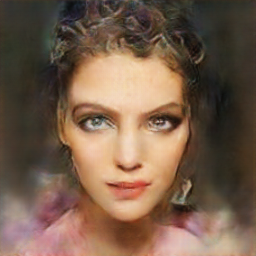

Supplement: Supplemental Information 5 [file peerj-cs-07-760-s005.zip › 04/221-targets-outputs.png]

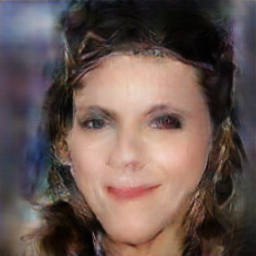

Supplement: Supplemental Information 5 [file peerj-cs-07-760-s005.zip › 04/222-targets-outputs.png]

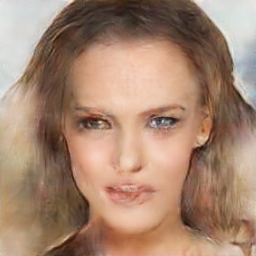

Supplement: Supplemental Information 5 [file peerj-cs-07-760-s005.zip › 04/223-targets-outputs.png]

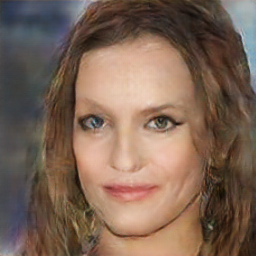

Supplement: Supplemental Information 5 [file peerj-cs-07-760-s005.zip › 04/224-targets-outputs.png]

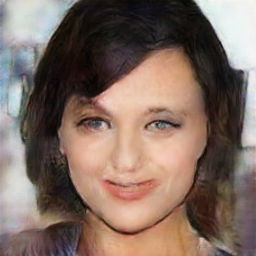

Supplement: Supplemental Information 5 [file peerj-cs-07-760-s005.zip › 04/225-targets-outputs.png]

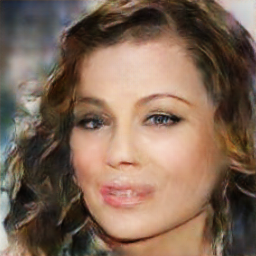

Supplement: Supplemental Information 5 [file peerj-cs-07-760-s005.zip › 04/226-targets-outputs.png]

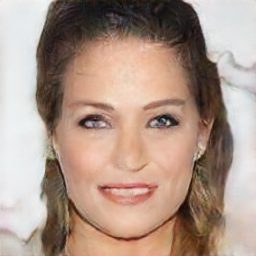

Supplement: Supplemental Information 5 [file peerj-cs-07-760-s005.zip › 04/227-targets-outputs.png]

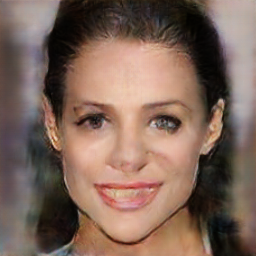

Supplement: Supplemental Information 5 [file peerj-cs-07-760-s005.zip › 04/228-targets-outputs.png]

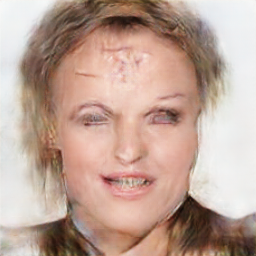

Supplement: Supplemental Information 5 [file peerj-cs-07-760-s005.zip › 04/229-targets-outputs.png]

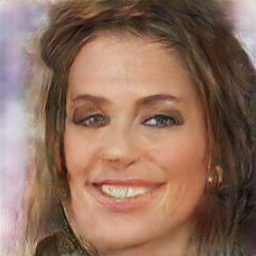

Supplement: Supplemental Information 5 [file peerj-cs-07-760-s005.zip › 04/230-targets-outputs.png]

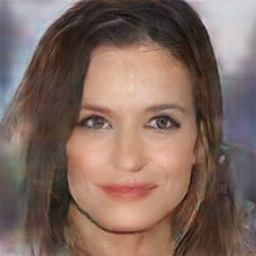

Supplement: Supplemental Information 5 [file peerj-cs-07-760-s005.zip › 04/231-targets-outputs.png]

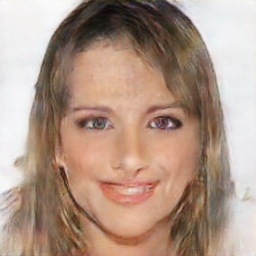

Supplement: Supplemental Information 5 [file peerj-cs-07-760-s005.zip › 04/232-targets-outputs.png]

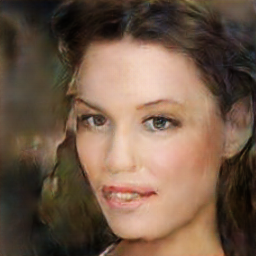

Supplement: Supplemental Information 5 [file peerj-cs-07-760-s005.zip › 04/233-targets-outputs.png]

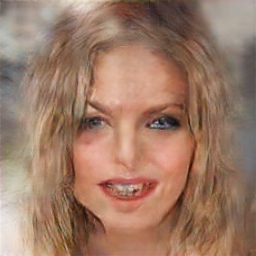

Supplement: Supplemental Information 5 [file peerj-cs-07-760-s005.zip › 04/234-targets-outputs.png]

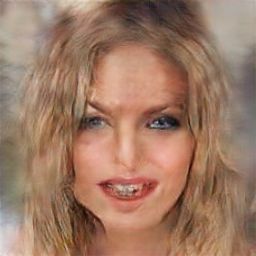

Supplement: Supplemental Information 5 [file peerj-cs-07-760-s005.zip › 04/235-targets-outputs.png]

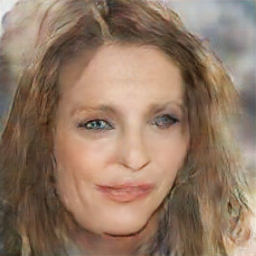

Supplement: Supplemental Information 5 [file peerj-cs-07-760-s005.zip › 04/236-targets-outputs.png]

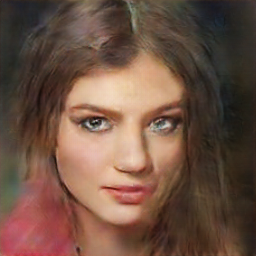

Supplement: Supplemental Information 5 [file peerj-cs-07-760-s005.zip › 04/237-targets-outputs.png]

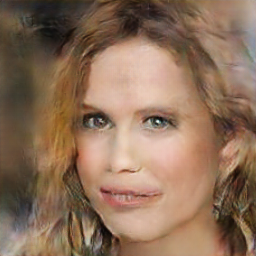

Supplement: Supplemental Information 5 [file peerj-cs-07-760-s005.zip › 04/238-targets-outputs.png]

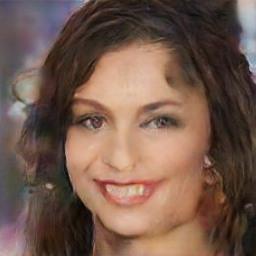

Supplement: Supplemental Information 5 [file peerj-cs-07-760-s005.zip › 04/239-targets-outputs.png]

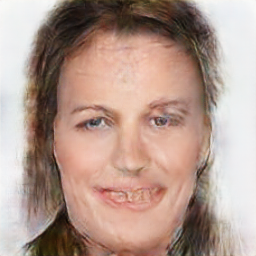

Supplement: Supplemental Information 5 [file peerj-cs-07-760-s005.zip › 04/240-targets-outputs.png]

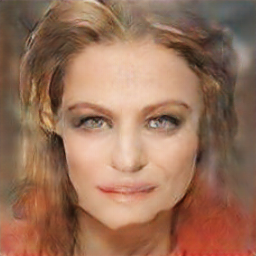

Supplement: Supplemental Information 5 [file peerj-cs-07-760-s005.zip › 04/241-targets-outputs.png]

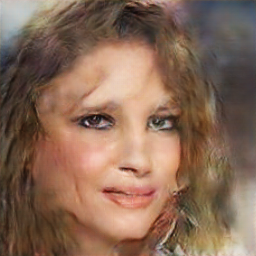

Supplement: Supplemental Information 5 [file peerj-cs-07-760-s005.zip › 04/242-targets-outputs.png]

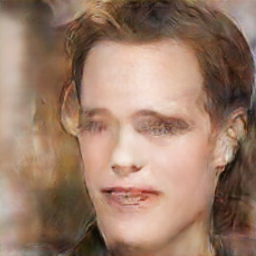

Supplement: Supplemental Information 5 [file peerj-cs-07-760-s005.zip › 04/243-targets-outputs.png]

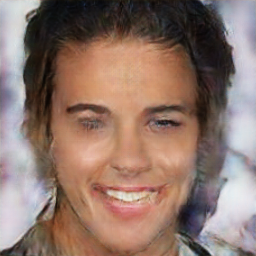

Supplement: Supplemental Information 5 [file peerj-cs-07-760-s005.zip › 04/244-targets-outputs.png]

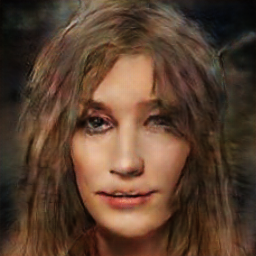

Supplement: Supplemental Information 5 [file peerj-cs-07-760-s005.zip › 04/245-targets-outputs.png]
