# Supplementary figures and images for: Small facial image dataset augmentation using conditional GANs based on incomplete edge feature input (part 6 of 6)
Source: PeerJ Comput Sci. 2021 Nov 17;7:e760. doi: 10.7717/peerj-cs.760 (PMC8627232; doi:10.7717/peerj-cs.760)

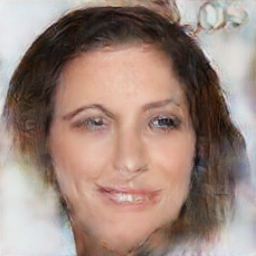

Supplement: Supplemental Information 5 [file peerj-cs-07-760-s005.zip › 06/246-targets-outputs.png]

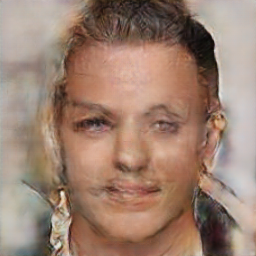

Supplement: Supplemental Information 5 [file peerj-cs-07-760-s005.zip › 06/247-targets-outputs.png]

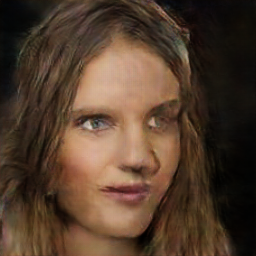

Supplement: Supplemental Information 5 [file peerj-cs-07-760-s005.zip › 06/248-targets-outputs.png]

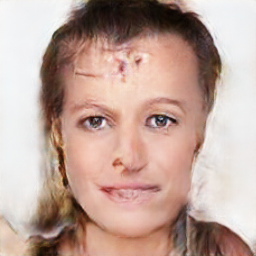

Supplement: Supplemental Information 5 [file peerj-cs-07-760-s005.zip › 06/249-targets-outputs.png]

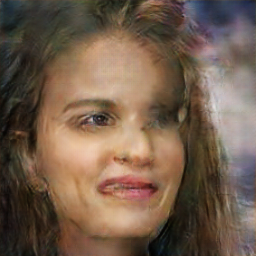

Supplement: Supplemental Information 5 [file peerj-cs-07-760-s005.zip › 06/250-targets-outputs.png]
