# Supplementary figures and images for: Small facial image dataset augmentation using conditional GANs based on incomplete edge feature input (part 5 of 6)
Source: PeerJ Comput Sci. 2021 Nov 17;7:e760. doi: 10.7717/peerj-cs.760 (PMC8627232; doi:10.7717/peerj-cs.760)

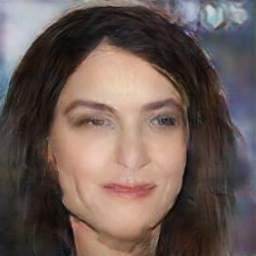

Supplement: Supplemental Information 5 [file peerj-cs-07-760-s005.zip › 04/246-targets-outputs.png]

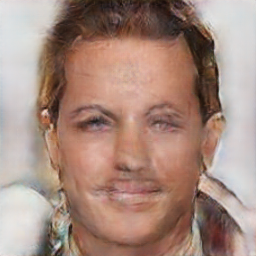

Supplement: Supplemental Information 5 [file peerj-cs-07-760-s005.zip › 04/247-targets-outputs.png]

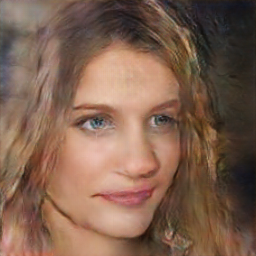

Supplement: Supplemental Information 5 [file peerj-cs-07-760-s005.zip › 04/248-targets-outputs.png]

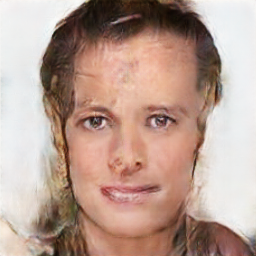

Supplement: Supplemental Information 5 [file peerj-cs-07-760-s005.zip › 04/249-targets-outputs.png]

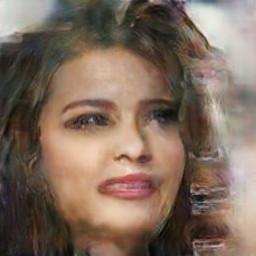

Supplement: Supplemental Information 5 [file peerj-cs-07-760-s005.zip › 04/250-targets-outputs.png]

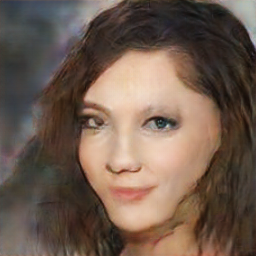

Supplement: Supplemental Information 5 [file peerj-cs-07-760-s005.zip › 05/201-targets-outputs.png]

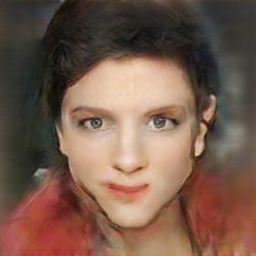

Supplement: Supplemental Information 5 [file peerj-cs-07-760-s005.zip › 05/202-targets-outputs.png]

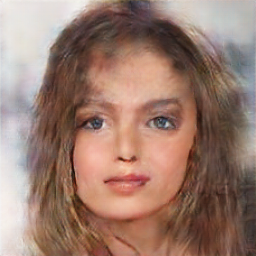

Supplement: Supplemental Information 5 [file peerj-cs-07-760-s005.zip › 05/203-targets-outputs.png]

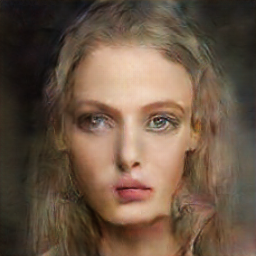

Supplement: Supplemental Information 5 [file peerj-cs-07-760-s005.zip › 05/204-targets-outputs.png]

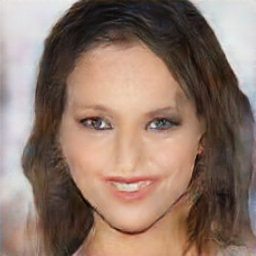

Supplement: Supplemental Information 5 [file peerj-cs-07-760-s005.zip › 05/205-targets-outputs.png]

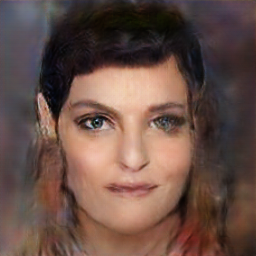

Supplement: Supplemental Information 5 [file peerj-cs-07-760-s005.zip › 05/206-targets-outputs.png]

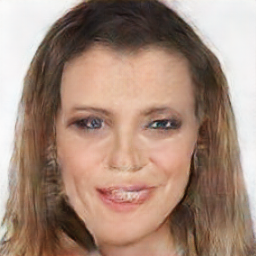

Supplement: Supplemental Information 5 [file peerj-cs-07-760-s005.zip › 05/207-targets-outputs.png]

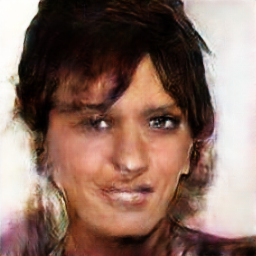

Supplement: Supplemental Information 5 [file peerj-cs-07-760-s005.zip › 05/208-targets-outputs.png]

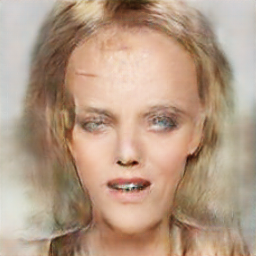

Supplement: Supplemental Information 5 [file peerj-cs-07-760-s005.zip › 05/209-targets-outputs.png]

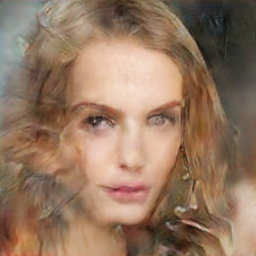

Supplement: Supplemental Information 5 [file peerj-cs-07-760-s005.zip › 05/210-targets-outputs.png]

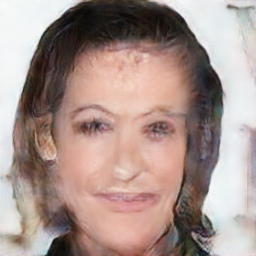

Supplement: Supplemental Information 5 [file peerj-cs-07-760-s005.zip › 05/211-targets-outputs.png]

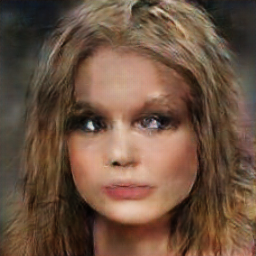

Supplement: Supplemental Information 5 [file peerj-cs-07-760-s005.zip › 05/212-targets-outputs.png]

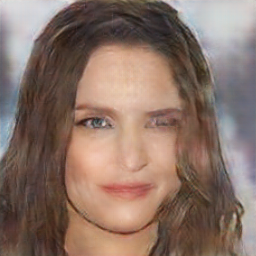

Supplement: Supplemental Information 5 [file peerj-cs-07-760-s005.zip › 05/213-targets-outputs.png]

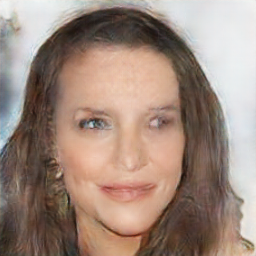

Supplement: Supplemental Information 5 [file peerj-cs-07-760-s005.zip › 05/214-targets-outputs.png]

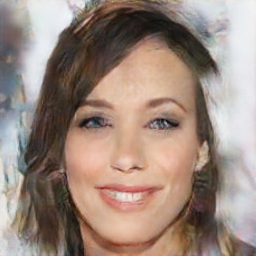

Supplement: Supplemental Information 5 [file peerj-cs-07-760-s005.zip › 05/215-targets-outputs.png]

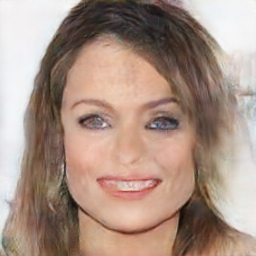

Supplement: Supplemental Information 5 [file peerj-cs-07-760-s005.zip › 05/216-targets-outputs.png]

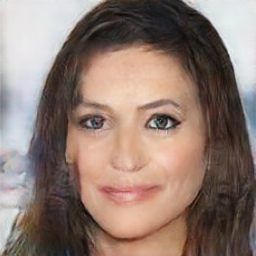

Supplement: Supplemental Information 5 [file peerj-cs-07-760-s005.zip › 05/217-targets-outputs.png]

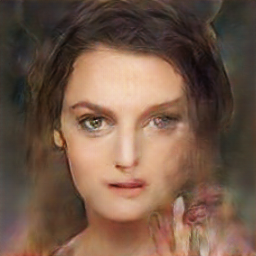

Supplement: Supplemental Information 5 [file peerj-cs-07-760-s005.zip › 05/218-targets-outputs.png]

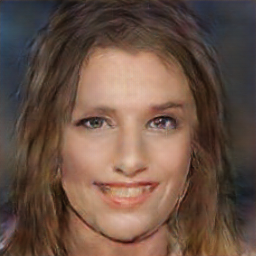

Supplement: Supplemental Information 5 [file peerj-cs-07-760-s005.zip › 05/219-targets-outputs.png]

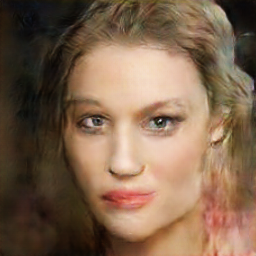

Supplement: Supplemental Information 5 [file peerj-cs-07-760-s005.zip › 05/220-targets-outputs.png]

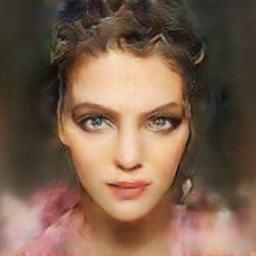

Supplement: Supplemental Information 5 [file peerj-cs-07-760-s005.zip › 05/221-targets-outputs.png]

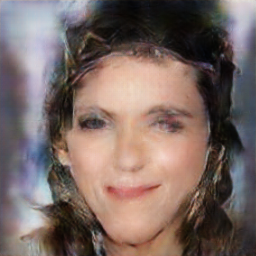

Supplement: Supplemental Information 5 [file peerj-cs-07-760-s005.zip › 05/222-targets-outputs.png]

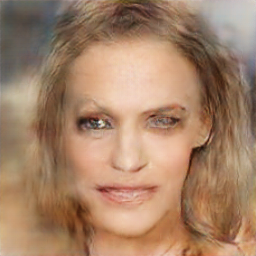

Supplement: Supplemental Information 5 [file peerj-cs-07-760-s005.zip › 05/223-targets-outputs.png]

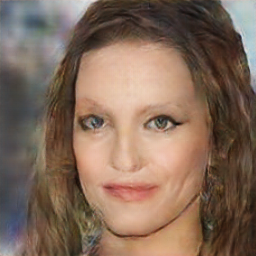

Supplement: Supplemental Information 5 [file peerj-cs-07-760-s005.zip › 05/224-targets-outputs.png]

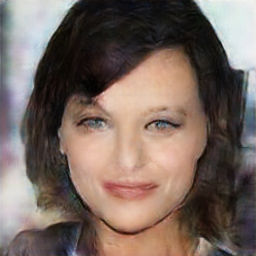

Supplement: Supplemental Information 5 [file peerj-cs-07-760-s005.zip › 05/225-targets-outputs.png]

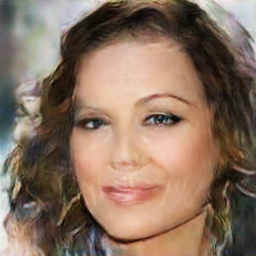

Supplement: Supplemental Information 5 [file peerj-cs-07-760-s005.zip › 05/226-targets-outputs.png]

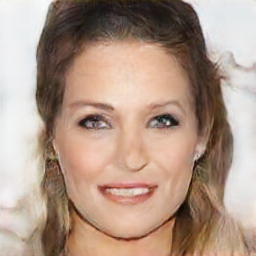

Supplement: Supplemental Information 5 [file peerj-cs-07-760-s005.zip › 05/227-targets-outputs.png]

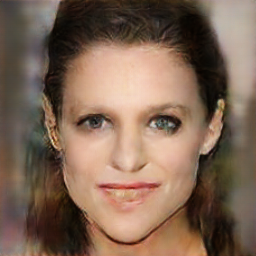

Supplement: Supplemental Information 5 [file peerj-cs-07-760-s005.zip › 05/228-targets-outputs.png]

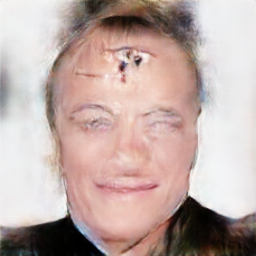

Supplement: Supplemental Information 5 [file peerj-cs-07-760-s005.zip › 05/229-targets-outputs.png]

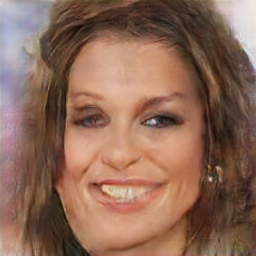

Supplement: Supplemental Information 5 [file peerj-cs-07-760-s005.zip › 05/230-targets-outputs.png]

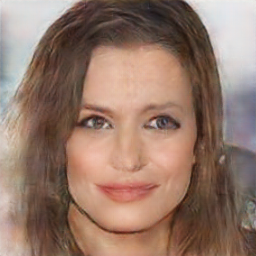

Supplement: Supplemental Information 5 [file peerj-cs-07-760-s005.zip › 05/231-targets-outputs.png]

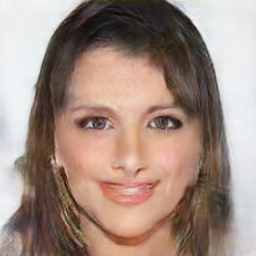

Supplement: Supplemental Information 5 [file peerj-cs-07-760-s005.zip › 05/232-targets-outputs.png]

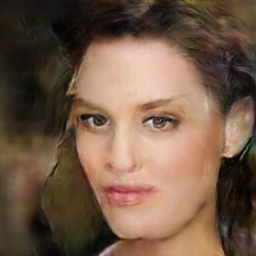

Supplement: Supplemental Information 5 [file peerj-cs-07-760-s005.zip › 05/233-targets-outputs.png]

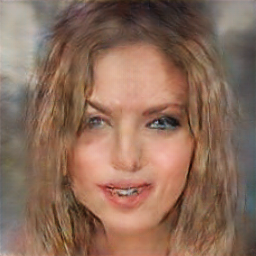

Supplement: Supplemental Information 5 [file peerj-cs-07-760-s005.zip › 05/234-targets-outputs.png]

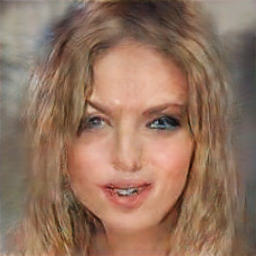

Supplement: Supplemental Information 5 [file peerj-cs-07-760-s005.zip › 05/235-targets-outputs.png]

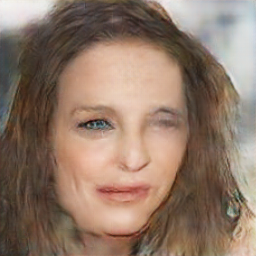

Supplement: Supplemental Information 5 [file peerj-cs-07-760-s005.zip › 05/236-targets-outputs.png]

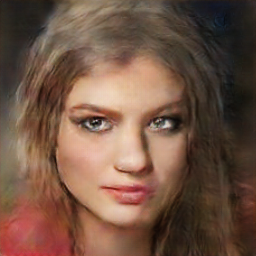

Supplement: Supplemental Information 5 [file peerj-cs-07-760-s005.zip › 05/237-targets-outputs.png]

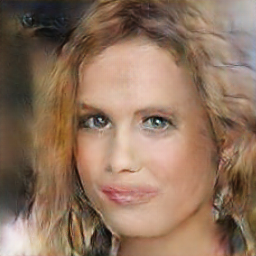

Supplement: Supplemental Information 5 [file peerj-cs-07-760-s005.zip › 05/238-targets-outputs.png]

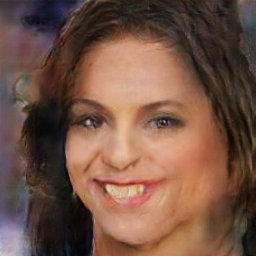

Supplement: Supplemental Information 5 [file peerj-cs-07-760-s005.zip › 05/239-targets-outputs.png]

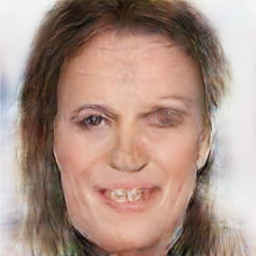

Supplement: Supplemental Information 5 [file peerj-cs-07-760-s005.zip › 05/240-targets-outputs.png]

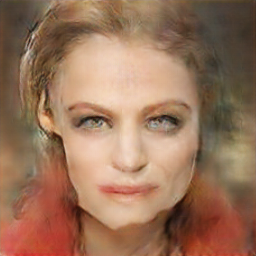

Supplement: Supplemental Information 5 [file peerj-cs-07-760-s005.zip › 05/241-targets-outputs.png]

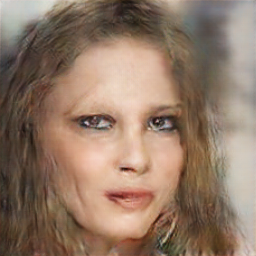

Supplement: Supplemental Information 5 [file peerj-cs-07-760-s005.zip › 05/242-targets-outputs.png]

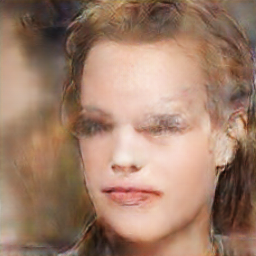

Supplement: Supplemental Information 5 [file peerj-cs-07-760-s005.zip › 05/243-targets-outputs.png]

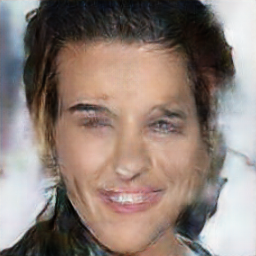

Supplement: Supplemental Information 5 [file peerj-cs-07-760-s005.zip › 05/244-targets-outputs.png]

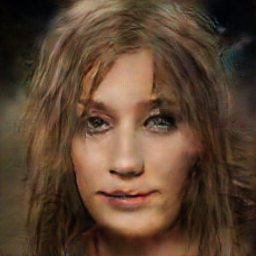

Supplement: Supplemental Information 5 [file peerj-cs-07-760-s005.zip › 05/245-targets-outputs.png]

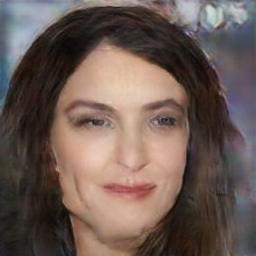

Supplement: Supplemental Information 5 [file peerj-cs-07-760-s005.zip › 05/246-targets-outputs.png]

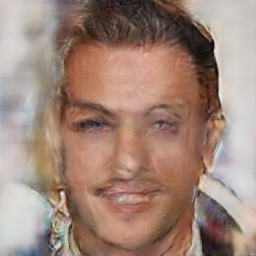

Supplement: Supplemental Information 5 [file peerj-cs-07-760-s005.zip › 05/247-targets-outputs.png]

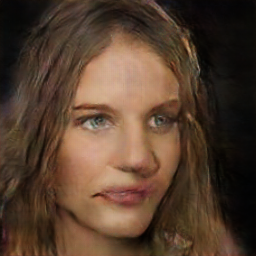

Supplement: Supplemental Information 5 [file peerj-cs-07-760-s005.zip › 05/248-targets-outputs.png]

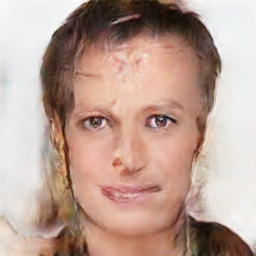

Supplement: Supplemental Information 5 [file peerj-cs-07-760-s005.zip › 05/249-targets-outputs.png]

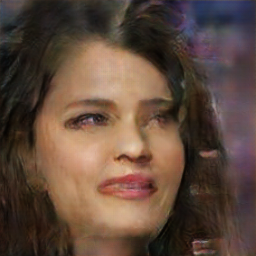

Supplement: Supplemental Information 5 [file peerj-cs-07-760-s005.zip › 05/250-targets-outputs.png]

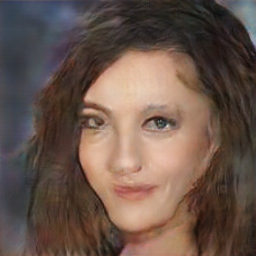

Supplement: Supplemental Information 5 [file peerj-cs-07-760-s005.zip › 06/201-targets-outputs.png]

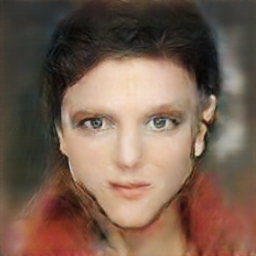

Supplement: Supplemental Information 5 [file peerj-cs-07-760-s005.zip › 06/202-targets-outputs.png]

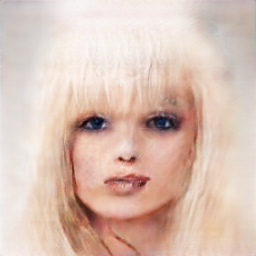

Supplement: Supplemental Information 5 [file peerj-cs-07-760-s005.zip › 06/203-targets-outputs.png]

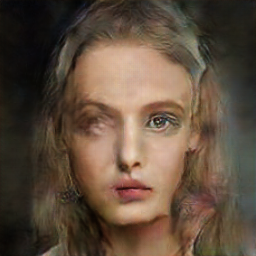

Supplement: Supplemental Information 5 [file peerj-cs-07-760-s005.zip › 06/204-targets-outputs.png]

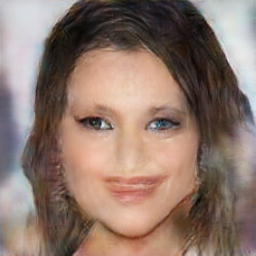

Supplement: Supplemental Information 5 [file peerj-cs-07-760-s005.zip › 06/205-targets-outputs.png]

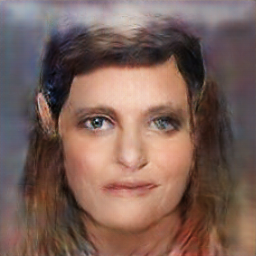

Supplement: Supplemental Information 5 [file peerj-cs-07-760-s005.zip › 06/206-targets-outputs.png]

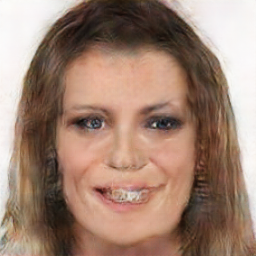

Supplement: Supplemental Information 5 [file peerj-cs-07-760-s005.zip › 06/207-targets-outputs.png]

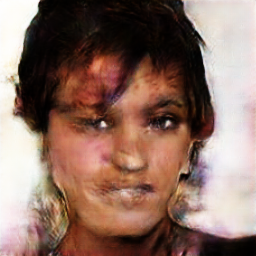

Supplement: Supplemental Information 5 [file peerj-cs-07-760-s005.zip › 06/208-targets-outputs.png]

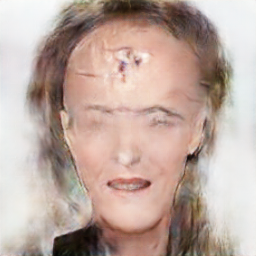

Supplement: Supplemental Information 5 [file peerj-cs-07-760-s005.zip › 06/209-targets-outputs.png]

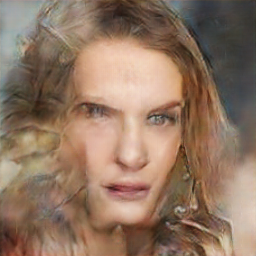

Supplement: Supplemental Information 5 [file peerj-cs-07-760-s005.zip › 06/210-targets-outputs.png]

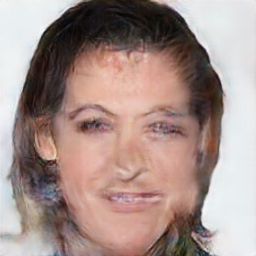

Supplement: Supplemental Information 5 [file peerj-cs-07-760-s005.zip › 06/211-targets-outputs.png]

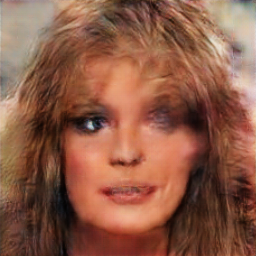

Supplement: Supplemental Information 5 [file peerj-cs-07-760-s005.zip › 06/212-targets-outputs.png]

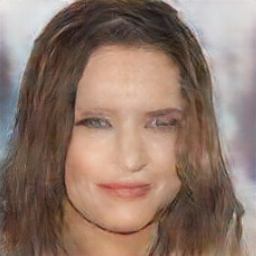

Supplement: Supplemental Information 5 [file peerj-cs-07-760-s005.zip › 06/213-targets-outputs.png]

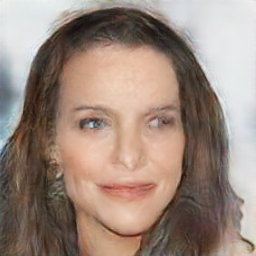

Supplement: Supplemental Information 5 [file peerj-cs-07-760-s005.zip › 06/214-targets-outputs.png]

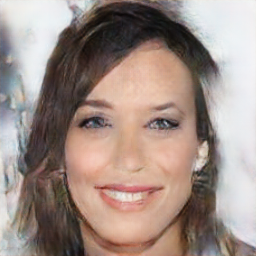

Supplement: Supplemental Information 5 [file peerj-cs-07-760-s005.zip › 06/215-targets-outputs.png]

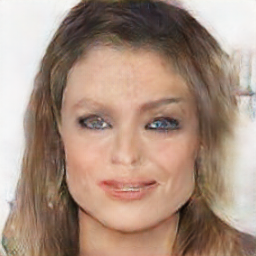

Supplement: Supplemental Information 5 [file peerj-cs-07-760-s005.zip › 06/216-targets-outputs.png]

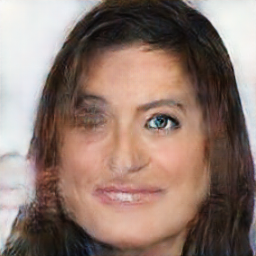

Supplement: Supplemental Information 5 [file peerj-cs-07-760-s005.zip › 06/217-targets-outputs.png]

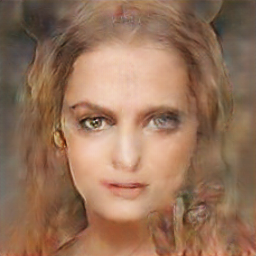

Supplement: Supplemental Information 5 [file peerj-cs-07-760-s005.zip › 06/218-targets-outputs.png]

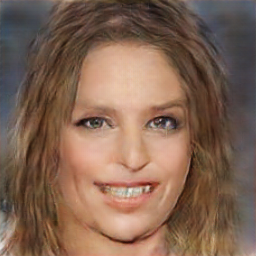

Supplement: Supplemental Information 5 [file peerj-cs-07-760-s005.zip › 06/219-targets-outputs.png]

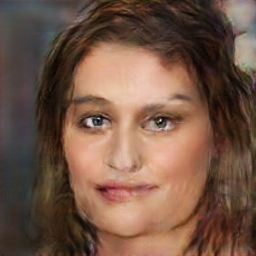

Supplement: Supplemental Information 5 [file peerj-cs-07-760-s005.zip › 06/220-targets-outputs.png]

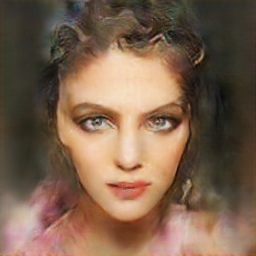

Supplement: Supplemental Information 5 [file peerj-cs-07-760-s005.zip › 06/221-targets-outputs.png]

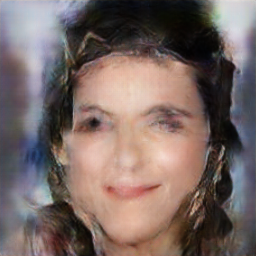

Supplement: Supplemental Information 5 [file peerj-cs-07-760-s005.zip › 06/222-targets-outputs.png]

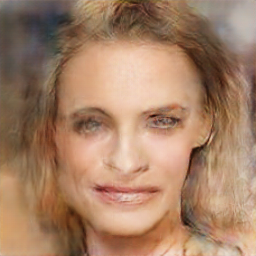

Supplement: Supplemental Information 5 [file peerj-cs-07-760-s005.zip › 06/223-targets-outputs.png]

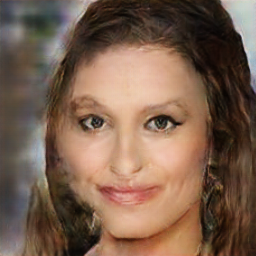

Supplement: Supplemental Information 5 [file peerj-cs-07-760-s005.zip › 06/224-targets-outputs.png]

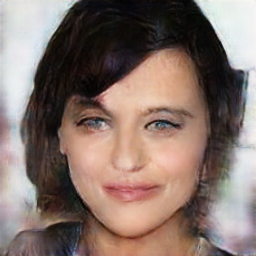

Supplement: Supplemental Information 5 [file peerj-cs-07-760-s005.zip › 06/225-targets-outputs.png]

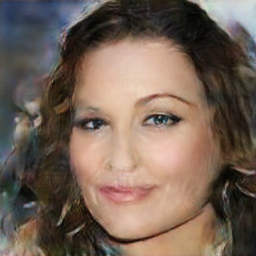

Supplement: Supplemental Information 5 [file peerj-cs-07-760-s005.zip › 06/226-targets-outputs.png]

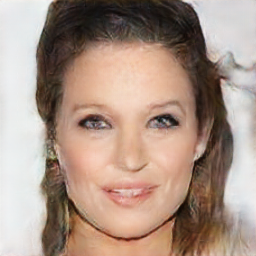

Supplement: Supplemental Information 5 [file peerj-cs-07-760-s005.zip › 06/227-targets-outputs.png]

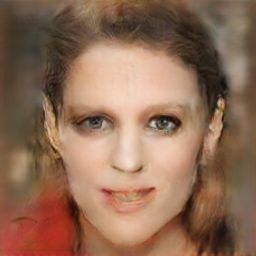

Supplement: Supplemental Information 5 [file peerj-cs-07-760-s005.zip › 06/228-targets-outputs.png]

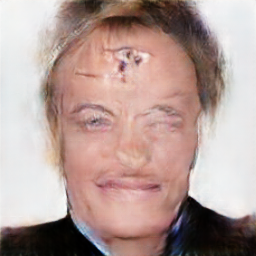

Supplement: Supplemental Information 5 [file peerj-cs-07-760-s005.zip › 06/229-targets-outputs.png]

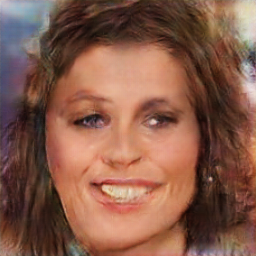

Supplement: Supplemental Information 5 [file peerj-cs-07-760-s005.zip › 06/230-targets-outputs.png]

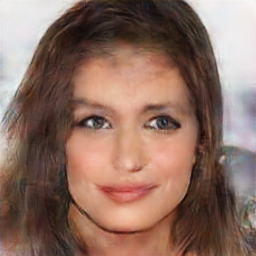

Supplement: Supplemental Information 5 [file peerj-cs-07-760-s005.zip › 06/231-targets-outputs.png]

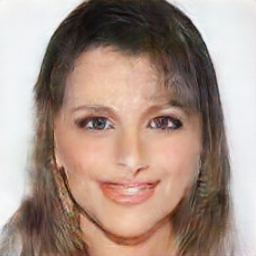

Supplement: Supplemental Information 5 [file peerj-cs-07-760-s005.zip › 06/232-targets-outputs.png]

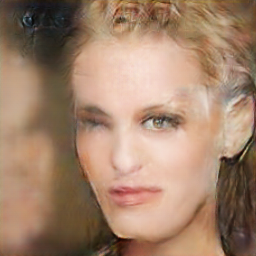

Supplement: Supplemental Information 5 [file peerj-cs-07-760-s005.zip › 06/233-targets-outputs.png]

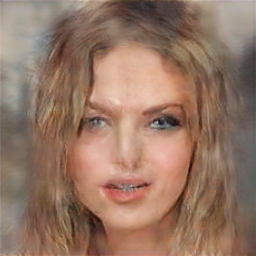

Supplement: Supplemental Information 5 [file peerj-cs-07-760-s005.zip › 06/234-targets-outputs.png]

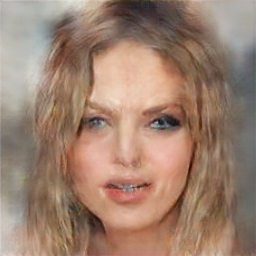

Supplement: Supplemental Information 5 [file peerj-cs-07-760-s005.zip › 06/235-targets-outputs.png]

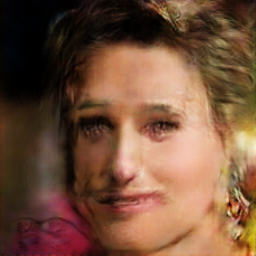

Supplement: Supplemental Information 5 [file peerj-cs-07-760-s005.zip › 06/236-targets-outputs.png]

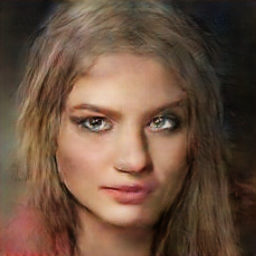

Supplement: Supplemental Information 5 [file peerj-cs-07-760-s005.zip › 06/237-targets-outputs.png]

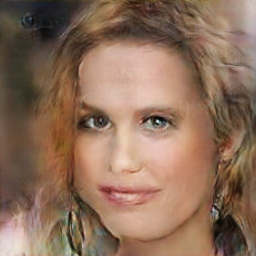

Supplement: Supplemental Information 5 [file peerj-cs-07-760-s005.zip › 06/238-targets-outputs.png]

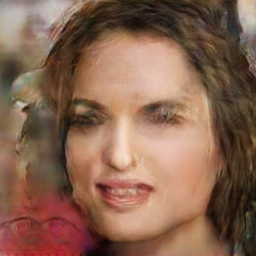

Supplement: Supplemental Information 5 [file peerj-cs-07-760-s005.zip › 06/239-targets-outputs.png]

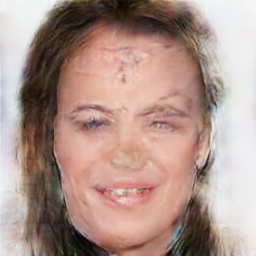

Supplement: Supplemental Information 5 [file peerj-cs-07-760-s005.zip › 06/240-targets-outputs.png]

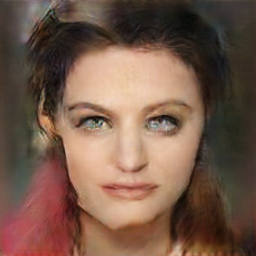

Supplement: Supplemental Information 5 [file peerj-cs-07-760-s005.zip › 06/241-targets-outputs.png]

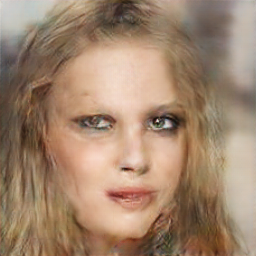

Supplement: Supplemental Information 5 [file peerj-cs-07-760-s005.zip › 06/242-targets-outputs.png]

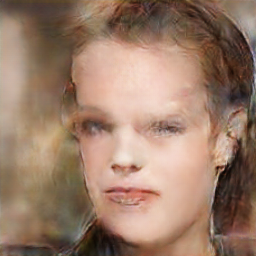

Supplement: Supplemental Information 5 [file peerj-cs-07-760-s005.zip › 06/243-targets-outputs.png]

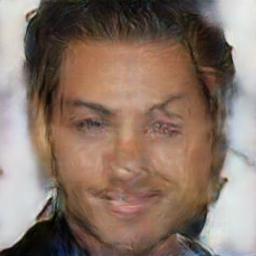

Supplement: Supplemental Information 5 [file peerj-cs-07-760-s005.zip › 06/244-targets-outputs.png]

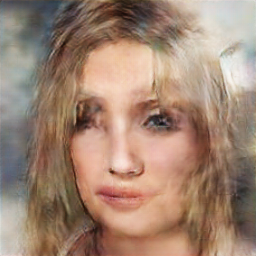

Supplement: Supplemental Information 5 [file peerj-cs-07-760-s005.zip › 06/245-targets-outputs.png]
